# Microbial Community Drivers in Anaerobic Granulation at High Salinity

**DOI:** 10.3389/fmicb.2020.00235

**Published:** 2020-02-26

**Authors:** Maria Cristina Gagliano, Dainis Sudmalis, Ruizhe Pei, Hardy Temmink, Caroline M. Plugge

**Affiliations:** ^1^Laboratory of Microbiology, Wageningen University & Research, Wageningen, Netherlands; ^2^Wetsus – European Centre of Excellence for Sustainable Water Technology, Leeuwarden, Netherlands; ^3^Department of Environmental Technology, Wageningen University & Research, Wageningen, Netherlands

**Keywords:** granular sludge, UASB, *Methanosaeta*, *Defluviitaleaceae*, filamentous microorganisms, EPS, fluorescence *in situ* hybridization, 16S rRNA gene sequencing

## Abstract

In the recent years anaerobic sludge granulation at elevated salinities in upflow anaerobic sludge blanket (UASB) reactors has been investigated in few engineering based studies, never addressing the microbial community structural role in driving aggregation and keeping granules stability. In this study, the combination of different techniques was applied in order to follow the microbial community members and their structural dynamics in granules formed at low (5 g/L Na^+^) and high (20 g/L Na^+^) salinity conditions. Experiments were carried out in four UASB reactors fed with synthetic wastewater, using two experimental set-ups. By applying 16S rRNA gene analysis, the comparison of granules grown at low and high salinity showed that acetotrophic *Methanosaeta harundinacea* was the dominant methanogen at both salinities, while the dominant bacteria changed. At 5 g/L Na^+^, cocci chains of *Streptoccoccus* were developing, while at 20 g/L Na^+^ members of the family *Defluviitaleaceae* formed long filaments. By means of Fluorescence *in Situ* Hybridization (FISH) and Scanning Electron Microscopy (SEM), it was shown that aggregation of *Methanosaeta* in compact clusters and the formation of filaments of *Streptoccoccus* and *Defluviitaleaceae* during the digestion time were the main drivers for the granulation at low and high salinity. Interestingly, when the complex protein substrate (tryptone) in the synthetic wastewater was substituted with single amino acids (proline, leucine and glutamic acid), granules at high salinity (20 g/L Na^+^) were not formed. This corresponded to a decrease of *Methanosaeta* relative abundance and a lack of compact clustering, together with disappearance of *Defluviitaleaceae* and consequent absence of bacterial filaments within the dispersed biomass. In these conditions, a biofilm was growing on the glass wall of the reactor instead, highlighting that a complex protein substrate such as tryptone can contribute to granules formation at elevated salinity.

## Introduction

Granular sludge bed based technologies such as the upflow anaerobic sludge blanket (UASB) reactor (Lettinga et al., 1980) are considered the most cost-effective at industrial scale for high-rate anaerobic treatment of concentrated organic wastewater (van Lier et al., 2015). The characteristic phenomenon in this process is sludge granulation, i.e., granular-shaped sludge is spontaneously formed within the reactor (Narihiro et al., 2009). Granular sludge is a spherical biofilm, rich in extracellular polymeric substances (EPS), possessing all the microbial trophic groups to complete the anaerobic degradation of organic matter (Sekiguchi et al., 1998; Seviour et al., 2009).

Many industrial processes produce substantial amounts of saline wastewater (Giustinianovich et al., 2018), for which high-rate treatment via anaerobic biofilm-based technologies (such as UASB reactors) are the most interesting, because of a successful biomass immobilization and retention (Xiao and Roberts, 2010; Kobayashi et al., 2018). However, high salinity is regarded a stress factor that severely hampers the performance of biological systems, negatively affecting biomass retention (Hemmelmann et al., 2013; Fang et al., 2018). In sludge granulation, granule strength and stability is considerably reduced in presence of high concentrations of Na^+^, leading to displacement of the bridging Ca^2+^ ions within the EPS matrix (Lambert et al., 1975; Bruus et al., 1992; Ismail et al., 2010). As consequence, non-adapted granules disintegrate, causing biomass wash-out and deteriorating process performance (Rinzema et al., 1988; Vallero et al., 2003; Ismail et al., 2008). Still, utilization of salinity adapted biomass was successfully applied to overcome this issue and obtain strong aggregates/granules (Feijoo et al., 1995; Hierholtzer and Akunna, 2014; Gagliano et al., 2017; Sudmalis et al., 2018).

The aggregation of microorganisms is a key event for granule development (Ding et al., 2015), positively influenced by the cell surface hydrophobicity, which enhances cell-to-cell adhesion and thereby the formation of aggregates (Wilén et al., 2018). Some studies suggested that proteins as substrate can increase hydrophobicity, favoring granule formation and improving their stability (McSwain et al., 2005; Zhu et al., 2015a; Li et al., 2016). Microorganisms are also able to respond to environmental changes by modifying their EPS composition, and therefore their surface properties and adhesion ability (Ahimou et al., 2007).

On the other hand, anaerobic granules stability is strongly related to the presence of structured microbial clusters, mostly dominated by methanogens (El-Mamouni et al., 1997; Sekiguchi et al., 1999; Gonzalez-Gil et al., 2001; Satoh et al., 2007). This is particularly true for the filamentous methanogenic archaeon *Methanosaeta*, which during the initial development of granules firstly attach on precursors, and subsequently forms a 3D network in which other microorganisms are entrapped (Wiegant, 1988; Liu et al., 2003; Angenent et al., 2004). Granulation increases rapidly when *Methanosaeta* is present in UASB reactors, ensuring the preservation of the granular structure and an optimal methane production (Uemura and Harada, 1993; El-Mamouni et al., 1995, 1997; van Haandel et al., 2014). *Methanosaeta harundinacea* cells were shown to significantly enhance granulation and performance of UASB reactors (Li et al., 2015). Our group demonstrated that *M. harundinacea* is a key-microorganism to obtain compact granules in UASB reactors at low and high salinity conditions, when using a salt-adapted, *Methanosaeta*-rich inoculum (Gagliano et al., 2017, 2018; Sudmalis et al., 2018).

Previous research investigating UASB reactors operated at high salinity (Rinzema et al., 1988; Vallero et al., 2003; Ismail et al., 2008, 2010; Li et al., 2014; Aslan and Şekerdağ, 2015; Gagliano et al., 2017; Sudmalis et al., 2018) did not carefully address the microbial ecology and its importance in driving aggregation and granules formation.

In our previous work (Sudmalis et al., 2018) granulation from dispersed biomass, accompanied by stable UASB process performance, at Na^+^ concentrations of 5 and 20 g/L was demonstrated for the first time. Microscopic analyses coupled with fluorescent *in situ* hybridization (FISH) showed dominance of filamentous *Methanosaeta* in the formed granules. In the present study, the two experimental set-ups used to monitor granulation at low (5 g/L Na^+^) and high (20 g/L Na^+^) salinity where compared, with respect to aggregation performances, with two UASB reactors (20 g/L Na^+^) with or without proteinaceous substrate. By combining comparative analyses of bacterial and archaeal 16S rRNA gene (by means of next generation sequencing (NGS) and clone libraries), together with microscopy techniques (scanning electron microscopy (SEM) and fluorescence microscopy), the objective of this work was to obtain a comprehensive overview and understanding of *Methanosaeta* and possible other microbial key-players favoring granulation under saline conditions.

## Materials and Methods

### Reactor Operation and Performance

#### Inoculum

The start-up biomass originated from a full-scale UASB reactor from the Shell plant in Moerdijk (Netherlands). The sludge was adapted to a sodium concentration of ≈8 g Na^+^/L for more than 10 years, with acetic and benzoic acids as the main substrate (Jeison et al., 2008). Before the reactors inoculation, the biomass was dispersed by forcing it through a 125 μm sieve.

#### Granular Sludge Reactor Operation

In total, four double-walled glass UASB bioreactors were inoculated with 6g VSS/L of anaerobic inoculum. Reactors R1 and R2, with 0.7 L active volume and 1 L total volume, were operated for 217 days. Reactors R3 and R4, with 1.98 L active volume and 3.13 L total volume, were operated for 120 days. The reactors were running at 35 ± 1°C and fed with a synthetic wastewater consisting of macro and micro-nutrients (Sudmalis et al., 2018). In all reactors, the upflow velocity at final loading rate was 1 m/h, while the pH remained close to neutral throughout reactor operation. A summary of operational conditions and performances is shown in Table 1.

**TABLE 1:** The main operational parameters and performance of the four UASB reactors.

	**R1**	**R2**	**R3**	**R4**
Operating Temperature	35 ± 1°C	35 ± 1°C	35 ± 1°C	35 ± 1°C
Start-up Inoculum (g VSS/L)	6	6	6	6
Salinity	5g Na^+^/L	20g Na^+^/L	20g Na^+^/L	20g Na^+^/L
COD ratio D-glucose:acetate	3:2	3:2	3:2	3:2
COD proportion of protein substrate	Tryptone 1	Tryptone 1	Tryptone 1	Leucine 0.5* Proline 0.5
Loading rate during start-up	1 g COD/L⋅d	1 g COD/L⋅d	1 g COD/L⋅d	1 g COD/L⋅d
Increase of influent COD from 3g/L to 7g/L**	Day 31	Day 31	Day 26	Day 26
Increase of influent COD from 7g/L to 12g/L**	Day 52	Day 52	Day 60	Day 60
sCOD removal at the final loading (%)	98.4 ± 0.4	95.3 ± 1.5	94.2 ± 1.2	89.5 ± 3.0
Average Biogas Methane content	63.6 ± 4.6%	68.4 ± 4.3%	70 ± 2.9%	67.4 ± 4.4%
Total Experiment Time (days)	217	217	120	120

*On day 71 leucine was replaced with glutamic acid. **Increase of influent COD corresponds to increasing loading rates.

#### Salinity Effect Experiment

To evaluate the salinity effect on granules formation and process performances, reactor R1 was operated at 5 g Na^+^/L, while reactor R2 was operated at 20 g Na^+^/L. In both reactors, the substrate was composed of soluble COD (sCOD) D-glucose, acetate, and tryptone in a 3:2:1 COD ratio. The influent COD concentration was increased in steps to reach a final organic loading rate (OLR) of approximately 16 g COD/L⋅d.

#### Substrate Effect Experiment

To evaluate the influence of proteinaceous substrate (tryptone) on granulation, reactor R3 was fed with the same substrate as described in the previous experiment, while in reactor R4 tryptone was substituted by the amino acids proline and leucine from day 0 to day 70, and by proline and glutamic acid from day 70 onward. The influent COD concentration was increased in steps to reach a final OLR of approximately 8.8 g COD/L⋅d.

#### Analytical Procedures

Daily biogas production was monitored with μFlow gas flow meter (Bioprocess Control, Sweden). Biogas composition was measured periodically as described in Steinbusch et al. (2008). Volatile fatty acids (VFAs) were quantified in the soluble fraction of effluent samples as described in Sudmalis et al. (2018). COD measurements were performed with LCK314, LCK514 and LCK1414 kits (HACH GMBH, Germany) on total (tCOD), colloidal (cCOD) and soluble fractions (sCOD) as described in Sudmalis et al. (2018).

### Microscopy Observation of Granules and Staining of the EPS Matrix

Freshly sampled granules were analyzed in time to follow the aggregation process by bright field microscopy using a Leica EZ 4D Stereomicroscope equipped with a Coach DSC webcam (Leica microsystems, Germany). Crystal violet 0.1% (v/v) staining was used to visualize the EPS layer on granules (O’Toole et al., 1999). Fluorescent staining of the protein portion of EPS was carried out by applying fluorescein isothiocyanate (FITC) (Thermo Fisher Scientific, United States) (final concentration of 0.5 g/L) on granules in their same reactor liquid-phase. After 30 min at RT in the dark, granules were washed with PBS/30% sacharose solution and observed through epifluorescence microscopy, as described in section “Fluorescent *in situ* Hybridization (FISH) and Microscopy Analysis.”

### Microbial Community Analysis

#### Sample Collection and DNA Extraction

Fresh granules were sampled at different time points from all reactors and stored at –20°C until DNA extraction. Genomic DNA was extracted from ≈ 500 mg of granules sampled from all reactors using a FastDNA^®^ SPIN kit for soil (MPBio, United States) according to the manufacturer’s instructions. Prior the DNA extraction, granules were washed with PBS 1X several times and pretreated by sonication (40 kHz, 50 W, 30 s). DNA concentration and purity were measured with the NanoDrop^®^ spectrophotometer (Thermo Fisher Scientific, Germany).

#### Amplification, Cloning and Sequencing of 16S rRNA Genes

16S rRNA genes were amplified with primers 27F (AGAGTTTGATCMTGGCTCAG) and 1369R (GCCCGGGAACGTATTCACCG) for Bacteria and primers 25F (CYGGTYGATYCTGCCRG) and 1386R (GCGGTGTGTGCAAGGAGC) for Archaea, using the GoTaq DNA polymerase kit (Promega, United States). PCR amplification of 16S rRNA genes of Archaea was carried out as described in Borrel et al. (2012). PCR reaction conditions for bacterial 16S rRNA genes were pre-denaturation at 94°C for 10 min, 30 cycles of denaturation at 94°C for 30 s, annealing at 52°C for 40 s, elongation at 72°C for 3 min and post-elongation at 72°C for 10 min. Cloning of purified PCR products was performed using pGEM-T Easy Vector System into *E. coli* JM109 competent cells (Promega, United States) according to the manufacturer’s instructions. A total of 96 clones from each reactor were selected for 16S rRNA Sanger sequencing and data analysis, as described in Gagliano et al. (2017). The sequences were reconstructed and deposited in GenBank, and accession numbers are listed in Supplementary Tables S1–S4. The affiliation was determined by the SILVA version 132 16S reference database (Quast et al., 2013) and the closest relatives were checked through the NCBI Blast alignment tool^1^.

#### Next Generation Sequencing (Illumina Sequencing and Computational Analysis)

Universal primers based on the V3-V4 hypervariable region of the prokaryotic 16S rRNA genes were applied for simultaneous amplification of Bacteria and Archaea in the reactor samples. Details on primers and the protocol applied are reported in Takahashi et al. (2014). Sequencing of PCR products was conducted on an Illumina sequencing system using the MiSeq Reagent Kit v2 (Illumina Inc., United States), and the reads generated were processed (i.e., filtered, clustered, taxonomically assigned and aligned) using the QIIME pipeline version 1.9.1 (Caporaso et al., 2010). Further details on sequencing statistics and data analysis are reported in Supplementary Material and Supplementary Figure S1. For the data presented in this study, we considered the most significant operational taxonomic units (OTUs) per each sample out of the total number of sequences, setting a cut-off value of 1%. The sequences reported have been deposited in the European Nucleotide Archive (ENA) database (accession nos. ERR3237939, ERR3237938, ERR3237937, ERR3237936, ERR3237935, ERR3237934, ERR3237933, ERR3237932, and ERR3237931).

#### Fluorescent *in situ* Hybridization (FISH) and Microscopy Analysis

For the FISH analysis, granules from each of the UASB reactors were fixed with 37% formaldehyde (w/w) according to Amann et al. (1995). After fixation, granules were washed with PBS to remove the excess salinity, and then stored at −20°C in ethanol/PBS (1:1). Granules were gently crushed by flushing in 1ml syringe with a 0.7 mm diameter needle, before FISH analysis (Hugenholtz et al., 2002). To visualize the organization and clustering of microorganisms in high salinity granules (R2 reactor, 20 g/L Na^+^), FISH analysis was carried out on 10 μm slices obtained after cryosectioning, as described in Gagliano et al. (2018). All oligonucleotide probes applied, labeled with Cy3-red or Alexa488-green fluorophores, are listed in Supplementary Table S5. Oligonucleotide probes were selected on the basis of the collected 16S rRNA sequences and the probeBASE database (Greuter et al., 2016). Samples were examined using an epifluorescence microscope BX41 (Olympus, Japan) equipped with Infinity Camera (Lumenera corporation, Canada). For the image analysis, the FIJI software package (version1.51g, Wayne Rasband, NIH, Bethesda, MD, United States) was used (Schindelin et al., 2012).

### Scanning Electron Microscopy (SEM)

Freshly sampled granules were fixed, dehydrated and sputter coated with tungsten before analysis, as described in Ismail et al. (2010). For samples visualization, a Magellan 400 SEM (FEI Company, OR, United States) was used, at an acceleration voltage of 2 kV and a beam current of 6 pA at RT.

## Results

### Salinity Effect Experiment

#### Reactors Operation and Granules Formation

**FIGURE 1:**
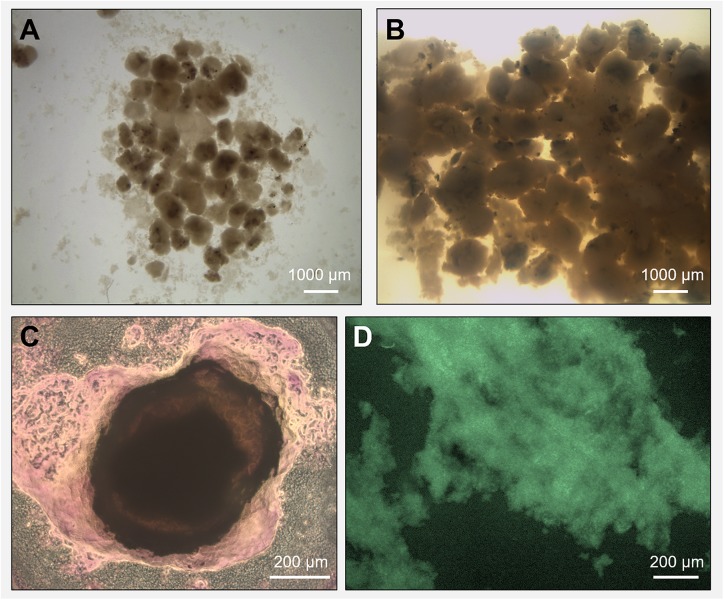
Microscopy observation of granules grown during the anaerobic process in reactor R1 (5 g/L Na^+^, in panel **A**) and reactor R2 (20 g/L Na^+^, in panel **B**). The extracellular polymeric substance (EPS) layer surrounding a high salinity granule from reactor R2 was visualized through crystal violet (total EPS, in panel **C**) and FITC (EPS proteins, in panel **D**) staining.

Biogas production and a high COD removal of >95% were achieved in both reactors R1 and R2 throughout the 217 days operation, in spite of the salinity level (Supplementary Figures S2A,B). The biogas was continuously produced in both R1 and R2 (Supplementary Figure S2C), with a net increase during the third phase of the process (with an OLR of 12 gCOD/L.d). A detailed description of the performance of these two reactors, as well as the granules formation dynamic are described in Sudmalis et al. (2018). Along the experimental period, the black grainy inoculum aggregated into granules with a whitish gel-like external EPS layer (Figures 1A,B), highlighted with crystal violet and FITC staining in Figures 1C,D. In reactor R2 (20 g/L Na^+^), the appearance of this whitish gel-layer was observed around day 80 (increase of influent COD), while 135 days of operation were needed to observe it on reactor R1 granules (5 g/L Na^+^). At the end of the process, visual and microscopic analysis evidenced that R2 granules were bigger than those developed in R1, and had a thicker EPS gel layer (Figures 1B,C). Thus, higher Na^+^ may have been beneficial for biomass aggregation, and the growth of this EPS layer can be seen as an adaptive response of the microbial community to the increased salinity.

#### Microbial Community Composition Using NGS and Clonal Analysis

**FIGURE 2:**
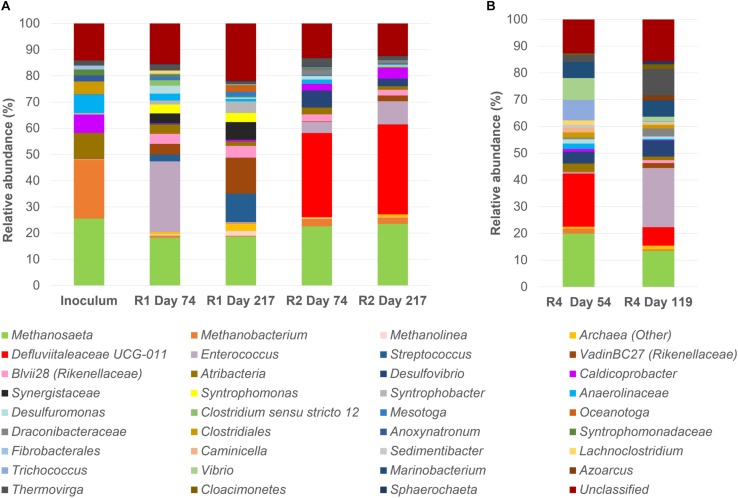
16S rRNA gene analysis by means of Next Generation Sequencing (NGS). Relative abundance of core operational taxonomic units (OTUs) (>1% relative abundance, 100% occupancy) and their taxonomy classification at the identified level (mostly genus level). All OTUs <1% are summed together and presented as unclassified. **(A)**: the start-up inoculum and samples taken from both reactor R1 (5 g/L Na^+^) and R2 (20 g/L Na^+^) at day 74 and day 217 (end of the reactor run). **(B)**: samples taken from R4 reactor (20 g/L Na^+^) at day 54 and day 119 (end of the reactor run).

A microbial community analysis by NGS was carried out on the initial inoculum sample and on granules taken from both reactors R1 and R2 at day 74 i.e., after the last increase of the OLR, and at the end of the operation period (day 217), and the results are reported in Figure 2. The community composition of reactor R1 and R2 at the end of the process changed significantly compared to the inoculum (Figure 2A).

In the start-up biomass, all of the most important bacterial groups were affiliated to the phylum *Firmicutes* (*Caldicoprobacter*, uncultured *Clostridiales* and *Anoxynatronum*) with the exception of the family *Anaerolinaceae* (7%) (Figure 2A). *Atribacteria* was the second most abundant bacterial phylum in the inoculum (10%). From this profile, two different bacterial populations developed in R1 (5 g/L Na^+^) and R2 (20 g/L Na^+^). At low salinity (5 g/L Na^+^), *Enterococcus* was the dominant genus at day 74 (27%), followed by members of the family *Rikenellaceae* (genera vadinBC27 at 4.08% and Blvii28 at 3.79%), *Synergistaceae*, *Atribacteria*, *Syntrophomonas, Desulfuromonas* and *Streptococcus* (Figure 2A). By the end of the experimental period (day 217), the family *Rikenellaceae* (vadinBC27 at 13.8% and Blvii28 at 4.4%), the genus *Streptococcus* (10.8%) and *Synergistaceae* (6.7%) increased and were the most abundant, with a stable presence of *Syntrophomonas* (3.5%) and the appearance of *Syntrophobacter* (4.5%) and *Oceanotoga* (2.4%) (Figure 2A). All the other main groups were decreasing or disappearing after 217 days of process. Most of the bacterial community members in reactor R2 (20 g/L Na^+^) were affiliated to the family *Defluviitaleaceae*, which relative abundance’s was slightly increasing, ranging from 32% at day 74 to 34% at the end of the digestion. An increasing trend was also observed for the other main OTUs, such as *Enterococcus*, *Caldicoprobacter* and vadinBC27 (*Rikenellaceae*) (Figure 2A). Members of *Atribacteria*, Blvii28 (*Rikenellaceae*) and *Draconibacteriaceae* were decreasing in relative abundance (Figure 2A).

Regarding the archaeal population, in the initial inoculum both the acetotrophic *Methanosaeta* (25.6%) and the hydrogenotrophic *Methanobacterium* (22.2%) were represented, while within R1 and R2 reactors, the dominance of *Methanosaeta* (18.7 and 23.6% at the end of the digestion period, respectively) over *Methanobacterium* (0.4 and 2.4% at the end of the digestion period, respectively) was evident, regardless the salinity levels.

To increase the accuracy and the resolution of the taxonomical classification, clonal analysis of 16S rRNA gene was carried out on samples taken at the end of the reactor operation, and the results are shown in Supplementary Tables S1, S2. The dominant Archaea in R1 and R2 granules had a high similarity (99% based on 16S rRNA) to *Methanosaeta harundinacea*, with 90 and 97% of the total clones, respectively (Supplementary Table S1). Just a few other identified and unidentified Archaea were detected (Supplementary Table S1). The bacterial 16S rRNA gene sequences obtained from the clonal analyses were closely related to *Rikenellaceae*, *Streptococcus* and *Synergistaceae* in reactor R1 and of *Defluviitaleaceae*, *Enterococcus* and *Clostridiales* in reactor R2, confirming the NGS results (Supplementary Table S2).

#### Microbial Community Observation by Microscopy

**FIGURE 3:**
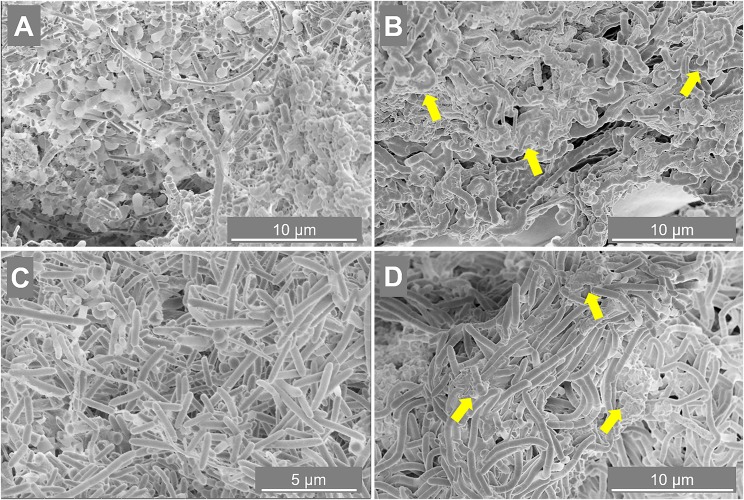
Scanning electron microscope (SEM) images of samples taken from reactor R1 operated at 5 g/L of Na^+^ **(A,B)** and reactor R2 operated at 20 g/L of Na^+^ **(C,D)**. The sampling was done at day 88 **(A,C)** and day 217 **(B,D)**. Yellow arrows indicate some spots more rich in extracellular polymeric substance (EPS).

To determine the importance of the microbial community in driving the granulation process at the two different salinities, SEM and FISH analysis were applied. Granules were sampled from both reactors at day 88 (appearance of EPS layers in R2 granules) and at day 217 (end of the reactor run), and observed through SEM. At day 88 (Figures 3A,C), in both reactors microorganisms mainly appeared as single cells, with few pairs and filaments. At day 217, granules where composed by tightly aggregated filaments, covered by a thick EPS layer (Figures 3B,D).

**FIGURE 4:**
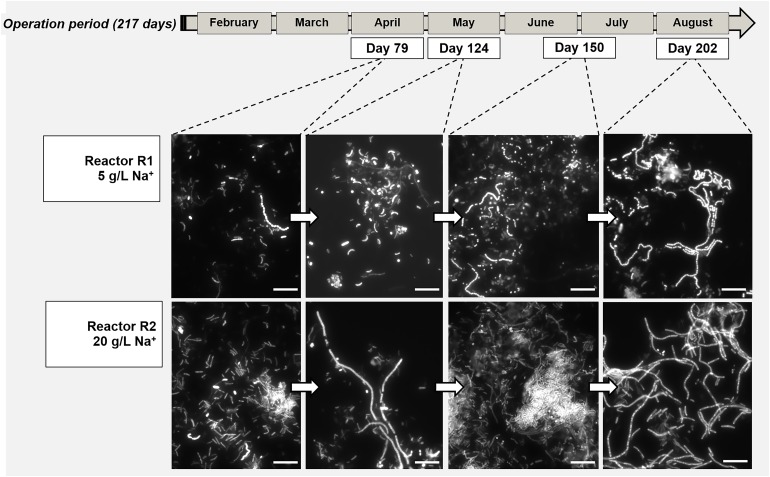
Evolution of bacterial aggregation in time during the digestion process surveyed by Fluorescence *in situ* hybridization (FISH), with the development of dominant populations of *Streptococcus* (Reactor R1, 5 g/L Na^+^) and *Defluviitaleaceae* (Reactor R2, 20 g/L of Na^+^). At both salinities, the filamentous bacteria emitted high fluorescence signal compared to the other community members. Size bar is 10 μm.

The morphology of the dominant filamentous microorganisms in the granules from both reactors was different: in R1, mostly twisted chains of coccus-shaped microorganisms were observed (Figure 3B), while in R2, filaments mainly consisted of aggregated rod shaped cells (Figure 3D). On the basis of the collected 16S rRNA sequences, the coccus- and rod-shaped morphologies were further identified by FISH analysis as *Streptococcus* sp. (Supplementary Figure S3A) and members of the family *Defluviitaleaceae* (Supplementary Figure S3B), respectively. FISH analysis and SEM are in agreement with NGS results (Figure 2), confirming both the increase in time of *Streptoccoccus* in reactor R1 and the dominance of *Defluviitaleaceae* in the community of reactor R2. The granule formation trend described in section “Reactors Operation and Granules Formation” as well as the cells assembly visualized by SEM, were further proven by following aggregation in time of the above mentioned bacterial morphologies by FISH. For R1, chains of *Streptococcus* cells were detected from June-July (day 135), while in R2 the rod-shaped *Defluviitaleaceae* cells were starting to cluster in April-May (from day 80 onward) (Figure 4).

**FIGURE 5:**
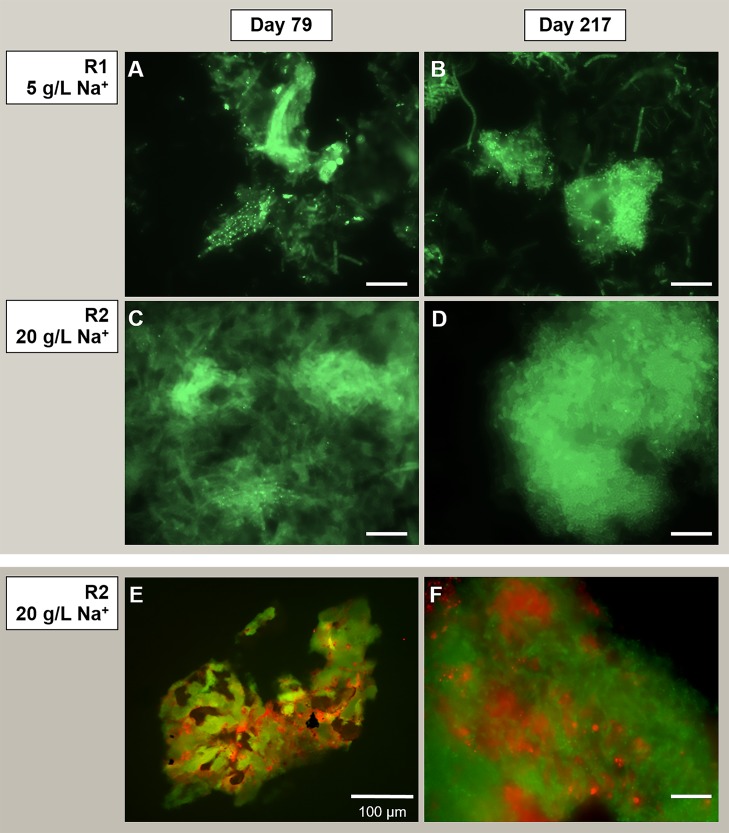
Observation of microbial aggregation within granules by Fluorescence *in situ* hybridization (FISH). From panels **(A–D)** the images show *Methanosaeta*-like clusters positive to the ARC915 probe (Archaea). The samples analyzed were taken at day 79 and day 217 from reactor R1 operated at 5 g/L of Na^+^ **(A,B)** and reactor R2 operated at 20 g/L of Na^+^ **(C,D)**. FISH was carried out also on cryosections from R2 reactor granules sampled at day 217 **(E,F)**, by applying both ARC915 (Archaea, in green) and EUB338 mix (Bacteria, in red) probes. Size bar is 10 μm or indicated otherwise.

On the other hand, the acetoclastic *Methanosaeta* was the most abundant aggregated microorganism in both granules from the initial stages of the process (Figures 5A,C), detected in both reactors in its round-shaped and “fibrous” clustered forms (an example in Figures 5A,B). When growing at 20 g/L Na^+^, the aggregation of these clusters was tighter than at 5 g/L Na^+^ (Figure 5). The dominance of *Methanosaeta* cells out of the total archaea was confirmed via FISH with ARC915, MSMX860 and MX825mix probes (see Supplementary Table S5 and Supplementary Material). FISH on cryosections (Figures 5E,F) showed the uniform distribution of *Methanosaeta* within the R2 granules and its structural role in establishing connections with bacterial clusters. The latter was clear also looking at the F_420_ autofluorescence emission of granules (Supplementary Figure S4), where the signal was uniformly distributed throughout the granule’s structure, with some dense spots of clustered cells.

### Substrate Effect Experiment

#### Reactor Operation and Biomass Granulation

In the second experiment reactors R3 and R4, both working at 20 g/L Na^+^, were fed either with or without tryptone, respectively (Table 1). Reactors performances were followed in terms of sCOD removal (Supplementary Figures S5A,B) and biogas production (Supplementary Figure S5C) throughout the reactors run. In the start-up period, when the OLR was around 1.4 g COD/L⋅day, the sCOD removal efficiencies in R3 and R4 were 81.9 ± 6.4 and 85.4 ± 6.2%, respectively. After day 24, with the gradual OLR increase to 7.5 g COD/L.d, in R3 the sCOD removal efficiency reached 92.5 ± 2.8%. In R4, where proline and leucine instead of tryptone were used as the carbon source (together with acetate and D-glucose), the sCOD removal was lower than in R3 (85.7 ± 1.0%). At day 71, leucine was replaced by glutamic acid, and the sCOD removal efficiency increased to 90.2 ± 2.5%. Biogas production was slightly higher for reactor R3, and this difference started to be evident just in the last phase of the digestion, at an OLR of 7.5 g COD/L⋅d (Supplementary Figure S5C). The increasing OLRs resulted in a higher VFA accumulation in R4 compared to R3 (Supplementary Table S6 and Supplementary Figure S5), most likely due to higher capacity of R3 to convert influent sCOD into methane compared to R4. In reactor R4, all of the leucine was converted to isovaleric acid, which disappeared after the switch to glutamic acid, the latter most likely converted into propionate (Supplementary Table S6).

**FIGURE 6:**
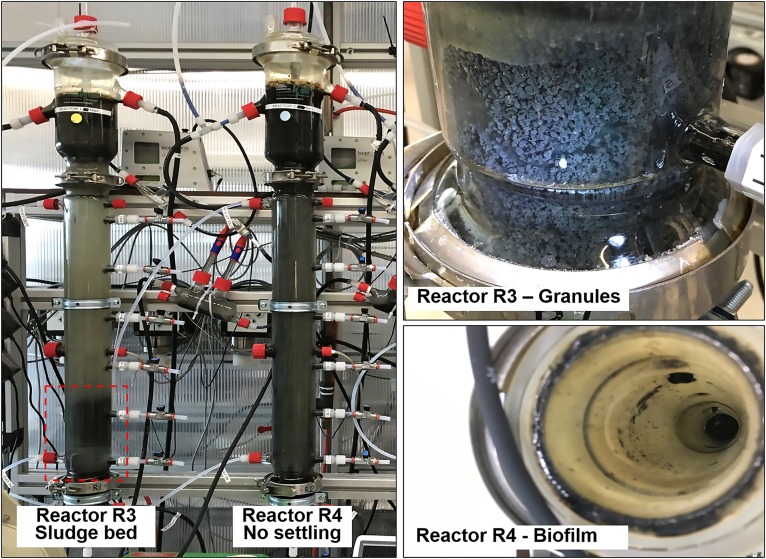
Comparison between reactor R3 (20 g/L of Na^+^, with tryptone), with an evident sludge bed (red box), and reactor R4 (20 g/L of Na^+^, with tryptone), were the biomass remained suspended and washed out. In reactor R3 granules formed, while in reactor R4 the biomass adhered onto the glass wall as biofilm.

Visual observation showed that granules occurred only in reactor R3, fed with tryptone as protein source (Figure 6). In reactor R3, the sludge bed started to form from day 23 onward, and the reactor was fully filled by granules after ≈40 days. Meanwhile, within 130 days of operation of R4, fed with proline and leucine, and from day 71 onward with proline and glutamic acid (as replacement of tryptone), no granular sludge formed, and a biofilm was growing on the glass wall of the reactor instead (Figure 6). This indicates that a complex proteinaceous substrate such as tryptone contributes to granules formation at high salinity conditions (20 g/L Na^+^).

#### Microbial Community Composition Using NGS and Clonal Analysis

In order to understand at the microbial level the lack of granulation in reactor R4, microbial community analysis using NGS of 16S rRNA was carried out on biomass sampled at day 54 and at the end of the operation period (day 119) (Figure 2B). A comparison between R3 and R4 microbial communities was achieved by clonal analysis, and the results are shown in Supplementary Material (Supplementary Tables S1, S3, S4). Similar to reactors R1 and R2, *Methanosaeta* was the dominant methanogen detected at the end of the process in R3 and R4 (Supplementary Table S1). However, considering the NGS data presented in Figure 2B, in reactor R4 the relative abundance of *Methanosaeta* decreased from 20% (Day 54) to 13.5% (end of the process). Hydrogenotrophic methanogens were represented by *Methanobacterium* (1.8%) at day 54, but these had disappeared at day 119 (Figure 2B). As observed for reactor R2 (20 g/L Na^+^), dominant bacteria in the R4 reactor at day 54 were members of *Defluviitaleaceae*, but their relative abundance decreased from 19.8% (Day 54) to 6.9% at the end of the process, with a concomitant appearance of *Enterococcus* (22% relative abundance) (Figure 2B). Other bacterial groups followed the same decreasing trend from day 54 to the end, as *Trichococcus, Vibrio* and *Atribacteria* (Figure 2B). On the other hand, the genus *Thermovirga* increased from 2.6% at day 54 to 10% at the end of the process. Changing the substrate from leucine to glutamic acid (at day 74) could have influenced the bacterial community composition in reactor R4.

**FIGURE 7:**
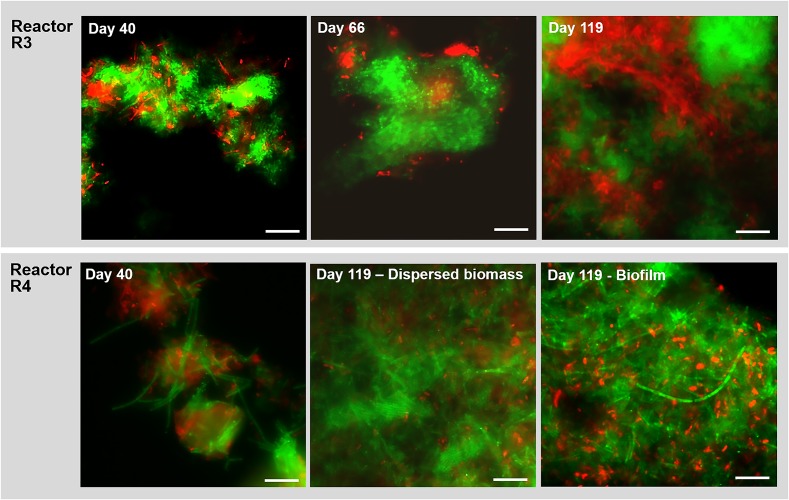
Observation of microbial aggregation within granules along time by Fluorescence *in situ* hybridization (FISH). Images show samples taken from reactor R3 (20 g/L of Na^+^, with tryptone) and R4 (20 g/L of Na^+^, without tryptone) at different time points. The sampling was done at day 40, day 66 and at the end of the digestion (day 119). FISH was carried out also on the biofilm sampled from the R4 reactor wall at day 119. The images show the ARC915 probe (Archaea) in green and the EUB338 mix probes (Bacteria) in red. Size bar is 10 μm.

The NGS results were confirmed by clonal analysis, as shown in Supplementary Table S4. In reactor R3, at day 54 *Defluviitaleaceae* (35%) and *Exiguobacterium* (20%) were the dominant bacteria, followed by *Enterococcus* (9.8%), *Synergistaceae*, *Atribacteria* and *Marinobacterium* (Supplementary Table S4). At the end of the process, *Exiguobacterium* drastically reduced to 1% relative abundance, while *Defluviitaleaceae*, *Enterococcus* and *Synergistaceae* were stable (Supplementary Table S4), differently than what was observed for reactor R4 (Figure 7 and Supplementary Table S4).

#### Microbial Community Observation by FISH

In reactor R3 (fed with tryptone), after 40 days of process and the appearance of granules, the aggregated form of *Methanosaeta* was detected (Figure 7). This aggregation became more compact from day 66 onward, with tight clusters of short filaments of different shapes (Figure 7), which were observed until the end of the experiment (Figure 7, day 119). In reactor R4 (without tryptone), non-aggregated, long *Methanosaeta* filaments were present in the dispersed sludge at the beginning of the process (Figure 7, day 40), and just small aggregates with loosely clustered cells were detected throughout the experimental period. At the end of the process, a higher amount of aggregated, “fibrous” shaped clusters of *Methanosaeta*, was present in the dispersed biomass (Figure 7, day 119). Regarding the *Bacteria* domain, as was also observed for reactor R2 (20 g/L Na^+^) (Figure 4), in reactor R3 members of *Defluviitaleaceae* gradually changed from rod shaped cells into filaments (Supplementary Figure S6). In reactor R4, without tryptone addition, no members of this family were detected by FISH. At day 119, in reactor R4 no bacterial filaments were present in the dispersed sludge (Figure7E), but instead big aggregates and short chains of the dominant *Enterococcus* sp. had appeared (Supplementary Figure S7). In the biofilm growing on the R4 reactor walls, and sampled at the end of the experiment, *Methanosaeta* cells were aggregated more tightly than in the dispersed biomass, but filamentous bacteria were not detected (Figure 7, biofilm at day 119).

## Discussion

### Salinity and Substrate Influence on Microbial Community and EPS Producers

In R1 and R2 UASB reactors rapid granular sludge formation from dispersed biomass at 5 g Na^+^/L and even at 20 g Na^+^/L occurred. Considering the microbial composition of the start-up inoculum (Figure 2), it is clear that while the change in salinity was working as a selective pressure for the development of a different bacterial community, *Methanosaeta* population was not influenced. The key-players in this community changed in response to low (5 g/L) and high (20 g/L) sodium levels. In both cases, the microbial community produced a surrounding EPS layer, which was thicker at higher salinity (Figure 1). EPS gel layer not only functions as a protective barrier toward salinity stress (Decho and Gutierrez, 2017), but the high local concentration of polymers can produce a profitable osmotic pressure difference with the external environment (Seminara et al., 2012; Yan et al., 2017).

With tryptone as a co-substrate, metabolically diverse bacteria in the granules were involved in EPS production and granulation. In reactor R1 granules (5 g/L Na^+^) *Streptococcus* filaments were dominant (Figures 3, 4), and members of this species are reported as both peptone and carbohydrate fermenters, producing EPS (Tagg et al., 2011; Sanalibaba and Cakmak, 2016). The other dominant group of *Rikenellaceae*, together with *Syntrophobacter* (Figure 2), were positively related to anaerobic sludge granulation driven by quorum sensing mechanisms (Ma et al., 2018). At higher salinity (reactor R2 and R3, 20 g/L Na^+^), members of *Defluviitaleaceae* were prevailing (Figures 2, 4), with relative abundances of 34% in reactor R2 and 30% in reactor R3 at the end of the process. The lactic acid bacterium *Enterococcus*, known to synthesize EPS from sugars (Mostafa et al., 2009; Bhat and Bajaj, 2018), was the most abundant after *Defluviitaleaceae*.

When tryptone was replaced by single amino acids, the presence and persisting of *Defluviitaleaceae* in reactor R4 was negatively affected (Supplementary Table S3). Also taking the microscopic observations in section “Reactors Operation and Granules Formation” into account, it is evident that tryptone availability is a key factor to promote growth and maintenance of granule forming microorganisms such as *Defluviitaleaceae*. *Enterococcus* presence and abundance along time instead was registered in reactor R4, and this can be related to the EPS production and formation of a biofilm (Figure 7) rather than granules. This is in line to the relative hydrophilicity possessed by *E. faecium* in comparison to the microbial community members present in UASB reactors hydrophobic granules (Daffonchio et al., 1995). Other abundant bacterial groups detected in R4 (without tryptone) and previously associated with biofilms, rather than granules formation, were *Thermovirga* (Lenhart et al., 2014), *Desulfovibrio* (Heggendorn et al., 2018) and *Marinobacterium* (Erable et al., 2009).

### *Methanosaeta* Clustering and Bacterial Filaments Formation as Key Events in Granulation

The microscopy investigations clearly showed the importance of both *Methanosaeta* clusters and bacterial filaments. The role of *M. harundinacea* is in line with previous studies demonstrating its potential to improve sludge granulation in UASB reactors, both under fresh water (Satoh et al., 2007; Li et al., 2015) and high salinity levels (Gagliano et al., 2017; Sudmalis et al., 2018). In this study, *M. harundinacea* was shown to provide structure to the granules, as indicated by its uniform distribution (Figure 5E and Supplementary Figure S3). *Methanosaeta* cells in both granule types were aggregated in two sorts of clusters: round shaped, rich in rods, and fibrous, with short filaments approaching each other (Figure 5). The development of *Methanosaeta* compact clusters (Figures 5A–D) is in contrast to what is usually observed under mesophilic, non-saline conditions, where *Methanosaeta* filaments are suspected to drive granulation (Zheng et al., 2006; Li et al., 2015), as described by the “*spaghetti theory*” (Wiegant, 1988; Liu et al., 2003). In this study, granules in reactor R1 and R2 increased in size over time when filamentous bacteria as *Streptococcus* and *Defluviitaleaceae* were gradually aggregating around the *Methanosaeta* clusters (Figures 3, 4). This microbial arrangement is similar to the one observed inside granules exposed to thermophilic (stress) conditions, where long filaments of *Methanosaeta* were replaced by dispersed cells forming microcolonies (Uemura and Harada, 1993; Syutsubo et al., 1997; Sekiguchi et al., 1999). In these thermophilic granules, filamentous bacteria where developing, improving granule settleability (Sekiguchi et al., 2001). Similarly, a salinity stress response in our UASB reactors could direct the aggregation toward the clustering of *Methanosaeta* and the filamentation of bacteria.

The aggregation properties of *Methanosaeta* likely rely on the peculiar glycoproteic sheet surrounding filaments and its hydrophobic characteristics (Beveridge et al., 1986; Pellerin et al., 1990; Nomura, 2012). Interestingly, fluorescence protein specific staining showed the presence of a proteinaceous outer layer not only on *M. harundinacea* cells (Supplementary Figure S8A), but also on *Defluviitaleaceae* (Supplementary Figures S8B,C). On the other hand, our previous studies applying lectin staining showed that *Streptococcus* chains in low salinity granules (5 g/L Na^+^) were covered by a capsular EPS rich in mannose (Gagliano et al., 2018). *Streptococcus* species showing a similar capsular EPS glycoconjugates pattern are known to promote bioaggregation (Tawakoli et al., 2017). Overall, *Methanosaeta* clusters were working as “granulation initiator,” hydrophobic nuclei on which *Streptococcus* and *Defluviitaleaceae* where aggregating as filaments, driving further the process by increasing the granules size in time (Figure 4).

Interestingly, with the lack of a complex mixture of proteins (tryptone) in the synthetic wastewater fed into reactor R4, the percentage of the granulation key players *Methanosaeta* and above all *Defluviitaleaceae* was drastically reduced in comparison to the other high salinity reactors (Figure 7 and Supplementary Table S4). Moreover, no active *Defluviitaleaceae* cells were detected by FISH at any time in reactor R4, either in dispersed biomass or in the biofilm. Cultured representatives of the family *Defluviitaleaceae* are thermophilic, anaerobic and sugar-fermentative bacteria (Jabari et al., 2012; Ma et al., 2017). Their presence in biogas plants and anaerobic processes has been demonstrated in several studies by 16S rRNA gene sequence diversity analysis (Fardeau et al., 2017). The species type of the family, *Defluviitalea saccharophila*, was isolated from an UASB reactor (Jabari et al., 2012). *Defluviitalea phaphyphila*, isolated from coastal sediment, is the only strain able to tolerate up to 5% NaCl (w/v), but not able to grow on proteins as casaminoacids (Ji et al., 2016). Thus, a new mesophilic species was represented in this study, with high salinity tolerance and most likely involved in protein fermentation and/or EPS production.

### Granules vs. Biofilm Growth: What About Proteins?

In this framework, above all tryptone, a mixture of peptides and amino acids resulting by the digestion of casein via trypsin protease, could be an essential substrate for granulation, as observed in R2 and R3 reactors (20 g/L of Na^+^) in connection to the growth of the filamentous *Defluviitaleaceae.* In general, casein hydrolysate positively influences EPS production (Jolly et al., 2002). Tryptone is an essential nitrogen source to culture lactic acid bacteria (LAB) for EPS production (Leroy and De Vuyst, 2016), and a potent growth enhancer for EPS producing *Bifidobacterium* strains (Poch and Bezkorovainy, 1991; Prasanna et al., 2012). Perhaps the lack of tryptone caused a lower EPS production, decreasing the granulation rate in reactor R4. A number of studies have demonstrated that casein hydrolysis can produce biologically active peptides promoting microbial growth (Phelan et al., 2009). For instance, the low-molecular-weight peptide fraction (<3 kDa) of casein hydrolysates was found to specifically promote the activity of different LAB enzymes leading to higher EPS production (Zhang et al., 2014). Interestingly, the trypsin hydrolysate of casein applied in the study of Zhang et al. (2007) was rich in glutamic acid, leucine and proline (0.843, 0.483, and 0.314 g kg^–1^ of casein hydrolysates, respectively). However, when these three amino acids where applied in this study as substrates in reactor R4, no granulation was observed, suggesting the importance of the entire peptide fraction to promote aggregation.

Besides the promotion of EPS production, protein availability as growth substrate can result in a diverse EPS secreted, with different chemical characteristics, influencing in turn the overall hydrophobicity of granules (Liu et al., 2004; Zhang et al., 2007). The latter is likely true, considering that in reactor R4 (without tryptone) part of the biomass formed a thick biofilm on the borosilicate glass inner reactor walls (Figure 6), a very hydrophilic surface. Thus, tryptone can also be considered a source of the building-blocks needed for the microbial synthesis of specific EPS that drives the cells aggregation toward the formation of spherical, rather than flat, biofilms. Some studies addressed the importance of proteins for aerobic granulation by modifying sludge surface properties, and found them to be essential for granules stability (McSwain et al., 2005; Xiong and Liu, 2013). Two recent investigations on anaerobic granules properties (Zhu et al., 2015b; Li et al., 2016) suggested that proteins are responsible for hydrophobicity, especially due to key components such as aromatic amino acids and their non-polar groups. Surface proteins with non-polar sites are known to be fundamental for cell attachment to hydrophobic substrata (Donlan, 2002) and herewith for self-aggregation. This could likely be the characteristic of the proteinaceous outer layer surrounding *Methanosaeta* and *Defluviitaleaceae* cells growing with tryptone (Supplementary Figure S8). A very recent study (Dubé and Guiot, 2019) found that *Methanosaeta* proteins were by far the most represented in the EPS extracted from three different granular anaerobic sludges, further proving its key role within the granules matrix.

## Concluding Remarks

Granulation of anaerobic biomass from dispersed inoculum was observed at Na^+^ concentrations of 5 and 20 g/L when feeding UASB reactors with glucose, acetate and tryptone as substrate. The salinity was acting as a selective pressure on the overall microbial community composition and on the main active bacteria (*Streptococcus* at 5 g/L Na^+^ and *Defluviitaleaceae* at 20 g/L Na^+^), while *M. harundinacea* was the dominant methanogen at both salinities. The two bacteria were growing as filaments and aggregating together with *Methanosaeta* clusters, shaping granules along time. The substitution of a complex protein substrate as tryptone with single amino acids (proline, leucine and glutamic acid) caused the lack of granules development, with a concomitant decrease in *Methanosaeta* and *Defluviitaleaceae* populations and the absence of compact clusters/filaments. Overall, both microbial community composition and substrate were shown to be important for anaerobic granulation at high salinity, an event yet to be fully explored in its complexity. Further insights into the metagenomics profiles and the protein expression patterns could further clarify the role of each member of the microbial community in terms of substrates utilization and EPS production, confirming a series of fundamental knowledge about microbial consortia applicability for high salinity wastewater treatment through UASB systems.

## Data Availability Statement

The datasets generated for this study can be found in the ERR3237939, ERR3237938, ERR3237937, ERR32 37936, ERR3237935, ERR3237934, ERR3237933, ERR323 7932, and ERR3237931.

## Author Contributions

MG, DS, HT, and CP conceived the study. DS designed, operated and monitored the laboratory-scale bioreactors together with RP. MG designed and operated the epifluorescence and confocal microscopy procedures, and performed the image analysis partly with RP. MG and DS performed the SEM analysis. MG extracted the DNA and analyzed the data from the NGS. MG and RP prepared the clonal libraries and analyzed the data. MG, DS, and RP interpreted the results. MG drafted the manuscript. DS, HT, CP, and MG revised the manuscript. All authors read and approved the final manuscript.

## Conflict of Interest

The authors declare that the research was conducted in the absence of any commercial or financial relationships that could be construed as a potential conflict of interest.

## Footnotes

1 https://blast.ncbi.nlm.nih.gov/Blast.cgi
