# Supplementary material for: Microbial Community Drivers in Anaerobic Granulation at High Salinity
Source: Front Microbiol. 2020 Feb 26;11:235. doi: 10.3389/fmicb.2020.00235 (PMC7054345; doi:10.3389/fmicb.2020.00235)
Supplement: Supplementary file 1 [file Data_Sheet_1.PDF]

frontiers

Supplementary Material

Microbial community drivers in anaerobic granulation at high salinity

M.C. Gagliano[1, 2, *, ∞], D. Sudmalis [3, ∞], R. Pei[2], H. Temmink[2, 3] and C.M. Plugge[1, 2]

[1] Laboratory of Microbiology, Wageningen University and Research, Stippeneng 4, 6708 WE, Wageningen, the Netherlands
[2] Wetsus – European Centre of Excellence for Sustainable Water Technology, Oostergoweg 9, 8911MA Leeuwarden, The Netherlands
[3] Department of Environmental Technology, Wageningen University and Research, Bornse Weilanden 9, 6708 WG, Wageningen, the Netherlands

[∞] *both authors contributed equally*

*** Correspondence:** Dr. M.Cristina Gagliano cristina.gagliano@wetsus.nl

Table S1 – Archaeal 16S rRNA gene clonal analysis of R1, R2, R3 and R4 UASB reactors at the end of each reactors run**.**

R1 Reactor

no. of clones	Accession number	Affiliation	Closest relative (accession no.)	Similarity (%)
85	MG062723	Uncultured *Methanosaeta*	*Methanosaeta harundinacea* 6Ac (CP003117.1)	99
1	MG062729	*Methanosaeta concilii*	*Methanosaeta concilii* strain GP6 (NR_102903.1)	99
5	MG062728	Uncultured *Methanobacteriales*	Uncultured archaeon clone ARCSB_122 (JQ738707.1)	99
	MG062726		Uncultured archaeon clone AP0-62 (KF564599.1)	99
3	MG062727	Uncultured *Methanolinea*	Uncultured *Methanolinea* sp. clone R1T2_A6 1(KX018771.1)	99
1	MG062725	Uncultured *Thermoplasmata*	Uncultured *Methanomassiliicoccales* archaeon clone: Alpha-MMA (LC200514.1)	99
95				

R2 Reactor

no. of clones	Accession number	Affiliation	Closest relative (accession no.)	Similarity (%)
92	MG062730	Uncultured *Methanosaeta*	*Methanosaeta* sp. HA (LC006969.1)	99
3	MG062724	Uncultured *Euryarchaeota*	Uncultured archaeon clone ARCSB_122 (JQ738707.1)	98
95				

R3 Reactor

no. of clones	Accession number	Affiliation	Closest relative (accession no.)	Similarity (%)
79	MN434996	Uncultured *Methanosaeta*	Uncultured *Methanosaeta* sp. clone D003011I03 (EU721747.1)	99
	MN434997	Uncultured *Methanosaeta*	Uncultured archaeon clone: AR80A32 (AB539923.1)	99
9	MG062724	Uncultured *Euryarchaeota*	Uncultured archaeon clone ARCSB_122 (JQ738707.1)	98
88				

R4 Reactor

no. of clones	Accession number	Affiliation	Closest relative (accession no.)	Similarity (%)
85	MN435003	Uncultured *Methanosaeta*	Uncultured *Methanosaeta* sp. clone D003011I03 (EU721747.1)	99
	MN435004		Uncultured *Methanosaeta harudinacea* clone S3_F10 (MG062730.1)	99
2	MN435001	Uncultured *Methanocalculus*	Uncultured archaeon clone PL-9C11 1(AY570669.1)	99
	MN435002		Uncultured *Methanocalculus* sp. clone Z3ALLARC44 (KX062751.1)	99
87				

Table S2 – Bacterial 16S rRNA gene clonal analysis of R1 and R2 UASB reactors after 217 days of digestion process.

R1 Reactor

no. of clones	Accession number	Affiliation (SILVA)	Closest relative (accession no.) (NCBI)	Similarity (%)
24	MK637498	Uncultured *Rikenellaceae*	Uncultured bacterium clone: Niigata-03 (AB243814.1)	99%
	MK637501		Uncultured bacterium clone ALM-360-38 (MH734889.1)	100%
20	MK637499	*Streptococcus* sp.	*Streptococcus henryi* strain OZK31 (KT716262.1)	99%
15	MK637493	Uncultured *Synergistaceae*	Uncultured *Synergistetes* bacterium clone: 3CP(-)_78 (AB908690.1)	99%
12	MK637506	Uncultured *Clostridia*	Uncultured organism clone MAT-CR-H4-A11 (EU245202.1)	89%
4	MK637494	Uncultured *Mesotoga*	Uncultured *Thermotogales* bacterium clone FC.C HM003079.1	99%
4	MK637496	*Sphaerochaeta* sp.	*Spirochaeta* sp. enrichment culture clone (KP178482.1)	99%
3	MK637495	Uncultured *Pelolinea*	Uncultured bacterium clone Nit5A0650_587 (FJ628288.1)	97%
2	MK637508	Uncultured *Syntrophobacter*	Uncultured *Syntrophobacter* sp. clone B16 (EU888828.1)	99%
1	MK637497	Uncultured *Latescibacteria*	Uncultured bacterium clone TDNP_Wbc97_110_1_21 (FJ516997.1)	96%
2	MK637509	Uncultured *Firmicutes*	Uncultured bacterium clone Er-LAYS-38 GU180170.1	99%
	MK637504		Uncultured bacterium clone 01f12 GQ132255.1	99%
1	MK637510	Uncultured *Propionibacteriales*	Uncultured bacterium clone D1 KJ808114.1	99%
88				

R2 Reactor

no. of clones	Accession number	Affiliation (SILVA)	Closest relative (accession no.) (NCBI)	Similarity (%)
48	MK637476	Uncultured *Defluviitaleaceae*	Uncultured bacterium clone H3098 (JX391238.1)	99%
13	MK637479	*Enterococcus* sp.	Enterococcus avium strain E6844 (NR_028748.1)	99%
10	MK637482	Uncultured *Clostridiales*	Uncultured bacterium clone DangY118-A25 (KC333944.1)	99%
5	MK637480	Uncultured *Rikenellaceae*	Uncultured Bacteroidetes/Chlorobi group bacterium clone De1156 (HQ183936.1)	99%
	MK637481		Uncultured Bacteroidetes bacterium clone RII-AN097 (JQ580476.1)	98%
4	MK637483	Uncultured *Synergistes*	Uncultured Synergistes sp. clone D004025G05 (EU721828.1)	98%
2	MK637489	Uncultured *Anaerovorax*	Uncultured bacterium clone NK-L29 (JN685456.1)	99%
6	MK637488	Uncultured bacterium	Uncultured OP9 bacterium clone QEDN10BG12 (CU927230.1)	99%
	MK637492		Uncultured bacterium clone P5-b21 (FR853013.1)	94%
1	MK637490	Uncultured *Geotoga*	Geotoga aestuarianus strain BPX-5 1 (MG696672.1)	99%
1	MK637478	*Sporolactobacillus* sp.	Sporolactobacillus nakayamae subsp. Racemicus (AB362636.1)	99%
1	MK637485	Uncultured *Atribacteria*	Uncultured bacterium clone De1870 (HQ184012.1)	99%
1	MK637486	*Sphaerochaeta* sp.	Spirochaeta sp. enrichment culture clone (KP178482.1)	99%
1	MK637487	Uncultured *Desulfuromonas*	Uncultured bacterium clone ALM-360-18 (MH734878.1)	99%
93				

Table S3– Bacterial 16S rRNA gene clonal analysis of R3 UASB reactor after 54 days and at the end of the reactors runs (day 119).

Day 54

no. of clones	Accession number	Affiliation (SILVA)	Closest relative (accession no.) NCBI	Similarity (%)
33	MN270919	Uncultured *Defluviitaleaceae*	Uncultured bacterium clone H3098 (JX391238.1)	99
21	MN270922	*Exiguobacterium* sp.	*Exiguobacterium* sp. Pb-WC11088 (JX913842.1)	100
9	MN270917	*Enterococcus* sp.	*Enterococcus avium* strain FDAARGOS_184 (CP024590.1)	100
5	MN270923	Uncultured *Atribacteria*	Uncultured bacterium clone De1870 (HQ184012.1)	99
4	MN270916	Uncultured *Marinobacterium*	Uncultured bacterium clone M2_56_F2 (JN683981.1)	100
4	MN270906	Uncultured *Clostridiales*	*Clostridia* bacterium P221(2) (GU370091.2)	96
4	MN270914	Uncultured *Synergistaceae*	Uncultured *Synergistetes* bacterium clone D121231H04 (GU180064.1)	98
3	MN270901	Uncultured *Rikenellaceae*	Uncultured *Bacteroidetes/Chlorobi* group bacterium clone De1156 (HQ183936.	98
3	MN270900	Uncultured *Trichococcus*	Uncultured bacterium clone RS-E76 (KC541372.1)	99
2	MN270920	Uncultured *Thermovirga*	Uncultured bacterium clone VHW_D_R9 (JQ085712.1)	100
1	MN270915	Uncultured *Lachnoclostridium*	*Clostridium amygdalinum* strain Marseille-P2095 (LT223651.1)	95
1	MN270913	Uncultured *Zixibacteria*	Uncultured organism clone SBZC_6039 (JN509545.1)	98
1	MN270908	Uncultured *Anaerolineaceae*	Uncultured bacterium clone Er-MLAYS-59 (EU542479.1)	97
1	MN270910	*Rhodococcus* sp.	*Rhodococcus* sp. NJ-530 (CP034152.1)	100
92				

Day 119

no. of clones	Accession number	Affiliation (SILVA)	Closest relative (accession no.) NCBI	Similarity (%)
28	MN413122	Uncultured *Defluviitaleaceae*	Uncultured *Clostridiales* bacterium clone Gran.bac.B09 1 (KX018762.1)	99
11	MN413128	*Enterococcus* sp.	*Enterococcus avium* strain FDAARGOS_184 (CP024590.1)	100
10	MN413123	Uncultured *Alkalibacter*	Uncultured *Alkalibacter* sp. clone De3229 (HQ183778.1)	99
8	MN413143	Uncultured *Synergistaceae*	Uncultured *Synergistes* sp. clone D004025G05 (EU721828.1)	98
7	MN413119	Uncultured *Clostridiales*	Uncultured *Clostridiales* bacterium clone Gran.bac.A05 (KX018760.1)	99
5	MN413124	Uncultured *Anaerolineaceae*	Uncultured organism clone MAT-CR-M7-G09 (GEU245894.1)	94
4	MN413144	Uncultured *Rikenellaceae*	Uncultured bacterium clone: Niigata-03 (AB243814.1)	99
3	MN413137	Uncultured *Mesotoga*	Uncultured *Thermotogales* bacterium clone FC.C (HM003079.1)	100
2	MN413129	Uncultured *Draconibacteriaceae*	Uncultured bacterium clone: 6T1cBa12 (LC183852.1)	99
2	MN413131	Uncultured *Lentimicrobiaceae*	Uncultured bacterium clone SSW62Au (EU592366.1)	99
2	MN413135	Uncultured *Christensenellaceae*	Uncultured bacterium clone DangY118-A25 (KC333944.1)	99
2	MN413118	Uncultured *Peptococcaceae*	Uncultured bacterium clone: 21ARB9 (AB668495.1)	99
2	MN413121	Uncultured *Desulfuromonadaceae*	Uncultured bacterium clone ALM-360-18 (MH734878.1)	99
1	MN270902	Uncultured *Exiguobacterium*	Uncultured bacterium clone: Niigata-24 (AB243992.1)	94
1	MN413125	Uncultured bacterium	Uncultured bacterium clone SA_58 (JQ738994.1)	94
1	MN413132	*Staphylococcus* sp.	*Staphylococcus pasteuri* strain JS7 (CP017463.1)	100
1	MN413145	Uncultured *Caldicoprobacteraceae*	Uncultured bacterium clone B19CH1_61_65 (HF558553.1)	95
1	MN413140	Uncultured *Acetoanaerobium*	Uncultured bacterium clone AB140 (KU667219.1)	98
1	MN413120	Uncultured *Spirochaetaceae*	*Spirochaeta* sp. enrichment culture clone (KP178482.1)	99
92				

Table S4– Bacterial 16S rRNA gene clonal analysis of R4 UASB reactor after 54 days and at the end of the digestion process (day 119).

Day 54

no. of clones	Accession number	Affiliation (SILVA)	Closest relative (accession no.) NCBI	Similarity (%)
40	MN414338	Uncultured *Defluviitaleaceae*	Uncultured bacterium clone JCC_RecOTU_41 (KX550184.1)	100
10	MN414330	*Trichococcus* sp.	Uncultured bacterium clone: 1, note: B2, *Carnobacterium* sp (AB495354.1)	100
8	MN414348	Uncultured *Atribacteria*	Uncultured bacterium clone De1870 (HQ184012.1)	99
5	MN414352	*Marinobacterium* sp.	*Marinobacterium* sp. IC961 strain: IC961 (AB196257.1)	100
5	MN414332	Uncultured *Clostridiaceae*	Uncultured bacterium clone Ll142-7F3 (FJ672758.1)	100
3	MN434991	Uncultured *Cloacimonetes*	Uncultured *Planctomycetales* bacterium clone B50-16 (KC555220.1)	97
3	MN414350	*Vibrio* sp.	*Vibrio cincinnatiensis* strain ATCC 35912 1 (NR_026122.1)	99
2	MN414337	Uncultured *Anaerolineaceae*	Uncultured bacterium clone Er-MLAYS-59 (EU542479.1)	97
2	MN414329	Uncultured *Sphaerochaeta*	Uncultured *Spirochaetes* bacterium clone D010011A22 (GU179806.1)	95
2	MN414346	Uncultured *Synergistaceae*	Uncultured *Synergistetes* bacterium clone D120231C06 (GU180061.1)	99
1	MN414331	Uncultured *Draconibacteriaceae*	Uncultured bacterium gene clone: 6T1cBa12 (LC183852.1)	99
1	MN414334	Uncultured *Caldicoprobacteraceae*	Uncultured bacterium clone B19CH1_61_65 (HF558553.1)	95
1	MN414336	Uncultured *Sedimentibacter*	Uncultured *Sedimentibacter* sp. clone Ced_Swamp_G3 (MG367117.1)	96
1	MN414339	Uncultured *Alkalibacter*	Uncultured bacterium gene clone: AS_B09 (LC214868.1)	100
1	MN414340	*Eggerthella* sp.	*Eggerthella lenta* strain AUH-Julong365 (JN874873.1)	100
1	MN414341	Uncultured *Peptococcaceae*	Uncultured bacterium clone Er-LAYS-38 1 (GU180170.1)	100
1	MN414343	Uncultured *Desulfuromonadaceae*	Uncultured bacterium clone ALM-360-18 (MH734878.1)	100
1	MN414345	Uncultured *Marinimicrobia*	Uncultured eubacterium AB16 (AF275926.2)	96
1	MN414335	Uncultured *Firmicutes*	Uncultured bacterium clone Er-MLAYS-91 (EU542485.1)	99
1	MN414342	Uncultured *Sulfurovum*	Uncultured bacterium clone GXU-03 (GU583971.1)	97
1	MN434990	Uncultured *Rhodococcus*	*Rhodococcus ruber* strain R1 (CP038030.1)	96
91				

(continue in the next page)

Day 119

no. of clones	Accession number	Affiliation (SILVA)	Closest relative (accession no.) NCBI	Similarity (%)
39	MN414182	*Enterococcus* sp.	*Enterococcus avium* strain FDAARGOS_184 (CP024590.1)	100
9	MN414190	Uncultured *Defluviitaleaceae*	Uncultured bacterium clone H3098 (JX391238.1)	99
8	MN414188	Uncultured *Synergistales*	Uncultured *Synergistes* sp. clone D004025G05 (EU721828.1)	99
4	MN434989	Uncultured *Cloacimonetes*	Uncultured *Planctomycetales* bacterium clone B50-16 (KC555220.1)	97
3	MN414180	Uncultured *Rikenellaceae*	Uncultured *Bacteroidetes* bacterium clone RII-AN097 (JQ580476.1)	98
3	MN414181	Uncultured *Sphaerochaeta*	Uncultured bacterium clone LC65 (FJ024715.1)	99
3	MN414185	*Rhodococcus* sp.	*Rhodococcus ruber* strain R1 (CP038030.1)	100
3	MN414187	Uncultured *Atribacteria*	Uncultured bacterium clone De1870 (HQ184012.1)	100
3	MN414198	Uncultured *Draconibacteriaceae*	Uncultured bacterium clone Asc-w-36 (EF632714.1)	97
2	MN414197	Uncultured *Marinobacterium*	Uncultured bacterium clone M2_56_F2 (JN683981.1)	96
2	MN414184	Uncultured *Piscirickettsiaceae*	Uncultured *Methylophaga* sp. clone DOM14 (HQ012276.1)	96
1	MN414196	Uncultured *Azoarcus*	Uncultured bacterium clone M1_146_B6 (JN683949.1)	99
1	MN414179	Uncultured *Clostridiales*	Uncultured bacterium isolate FBP407, clone 127A (LN680034.1)	99
1	MN434992	Uncultured *Desulfuromonas*	Uncultured bacterium clone ALM-360-18 1(MH734878.1)	99
1	MN414186	Uncultured *Lentimicrobiaceae*	Uncultured bacterium clone SSW62Au (EU592366.1)	93
1	MN414199	Uncultured bacterium	Uncultured Unclassified bacterium clone QEDS3AH04 (CU921882.1)	87
1	MN414193	Uncultured *Thermovirga*	Uncultured bacterium clone BA141 (AF323769.1)	99
85				

Table S5 - Oligonucleotide probe sequences, target microbial groups and stringency conditions used in this study.

<table>
<tr><th>Name</th><th>Target Group</th><th>Probe sequence (5'-3')</th><th>Formamide (%)</th><th>Reference</th></tr>
<tr><td>ARC915</td><td>Archaea</td><td>GTGCTCCCCCGCCAATTCCT</td><td>20 or 35</td><td>Stahl and Amann, 1991</td></tr>
<tr><td>EUB338</td><td>Bacteria</td><td>GCTGCCTCCCGTAGGAGT</td><td>20 or 35</td><td>Amann et al., 1990</td></tr>
<tr><td>CF319a</td><td>Bacteroidetes</td><td>TGGTCCGTGTCTCAGTAC</td><td>35</td><td>Manz et al., 1996</td></tr>
<tr><td>DELTA485A</td><td rowspan="3">Deltaproteobacteria</td><td>AGTTAGCCGGTGCTTCCT</td><td rowspan="3">35</td><td rowspan="3">Lücker et al., 2007</td></tr>
<tr><td>DELTA495B</td><td>AGTTAGCCGGCGCTTCCT</td></tr>
<tr><td>DELTA495C</td><td>AATTAGCCGGTGCTTCCT</td></tr>
<tr><td>CFX1223</td><td>Chloroflexi</td><td>CCATTGTAGCGTGTGTGTMG</td><td>35</td><td>Björnsson et al., 2002</td></tr>
<tr><td>SYN961</td><td>Synergystetes</td><td>GTTCTTCGGTTTGCATCG</td><td>20 or 35</td><td>Gagliano et al., 2015</td></tr>
<tr><td>STREPT</td><td>Streptococcaceae</td><td>CACTCTCCCCTTCTGCAC</td><td>30</td><td>Trebesius et al., 2000</td></tr>
<tr><td>LAC435</td><td>Lachnospiraceae [(1)]</td><td>TCTTCCCTGCTGATAGA</td><td>35</td><td>Kong et al., 2010</td></tr>
<tr><td>MSMX860</td><td>Methanosarcinales*</td><td>GGCTCGCTTCACGGCTTCCCT</td><td>45</td><td>Raskin et al., 1994</td></tr>
<tr><td>MG1200b</td><td>Methanomicrobiales</td><td>CRGATAATTCGGGGCATGCTG</td><td>20</td><td>Crocetti et al., 2006</td></tr>
<tr><td>LAB158</td><td>Lactobacilli-Enterococci</td><td>GGTATTAGCAYCTGTTTCCA</td><td>25</td><td>Harmsen et al. 1999</td></tr>
<tr><td>LGC354A</td><td rowspan="3">Firmicutes</td><td>TGGAAGATTCCCTACTGC</td><td rowspan="3">35</td><td rowspan="3">Meier et al. 1999</td></tr>
<tr><td>LGC354B</td><td>CGGAAGATTCCCTACTGC</td></tr>
<tr><td>LGC354C</td><td>CCGAAGATTCCCTACTGC</td></tr>
<tr><td>MX825</td><td>Methanosaetaceae**</td><td>TCGCACCGTGGCCGACACCTAGC</td><td>50</td><td>Raskin et al., 1994</td></tr>
<tr><td>MX825b</td><td rowspan="2">Methanosaetaceae subgroup**</td><td>TCGCACCGTTGCCGACACCTAGC</td><td>50</td><td>Crocetti et al., 2006</td></tr>
<tr><td>MX825c</td><td>TCGCACCGTGGCTGACACCTAGC</td><td>50</td><td>Crocetti et al., 2006</td></tr>
</table>

[(1)] (The probe was used to target *Defluviitaleaceae*. Sequences classified by the SILVA taxonomy as *Defluviitaleaceae* were also classified as *Lachnospiraceae* by the Greengenes taxonomy).

* (including *M.harundinacea*)

** (except *M.harundinacea*)

Materials and Methods

1. Next generation sequencing -QIIME 16S rRNA gene analysis

The paired-end MiSeq reads were merged based on the overlap between the two reads. Only merged sequences were used in subsequent analyses, and the non-overlapping pairs were discarded. The merged sequences were filtered, clustered, taxonomically assigned and aligned using the QIIME pipeline version 1.9.1 (Caporaso et al., 2010). The process consisted of quality checking, de-noising, and a microbial diversity analysis. The raw reads were cleaned-up from the primers sequences, and then placed in a single Fasta file using the script 'add_qiime_labels.py'. OTU picking was performed with the script 'pick_open_reference_otus.py' using the SILVA database version 128 (Quast et al., 2013) and the clustering method UCLUST (Edgar, 2010). The RDP classifier (version 2.2) (Wang et al., 2007) was used to classify the OTUs on the basis of the same SILVA database. With the QIIME script 'core_diversity_analyses.py', alpha- and beta-diversity statistics of the samples were calculated. The Illumina MiSeq sequencing of PCR products generated 708624 reads from seven samples (Fig. S1). The rarefaction curves were established towards a plateau after 55 000 reads were sequenced, indicating that sufficient coverage was obtained in this study (Fig. S1).

	Inoculum	R1 day 74	R1 day 217	R2 day 74	R2 day 217	R4 day 54	R4 day 119
**Sequences**	105577	96198	109079	116050	55405	105213	121102
**OTUs**	1275 ± 591.4	1513 ± 444	1526 ± 450	1249 ± 368	940 ± 273	1509 ± 446	1534 ± 453
**Core OTUs (occupancy = 100%, abundance> 1%)**	10	14	12	14	12	16	17

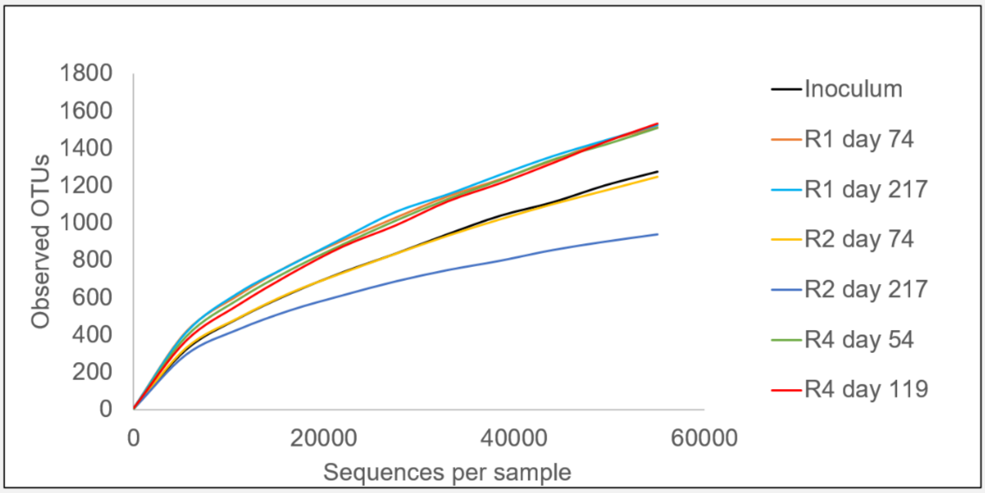

Fig.S1 – Detected OTUs, alpha diversity and rarefaction curves data obtained after amplicon sequencing.

2. FISH identification of *Methanosaeta harundinacea*

In this work, the inoculum used was characterized several times for the microbial composition, showing the presence and dominance of *M.harundinacea* (Gagliano et al., 2017; Sudmalis et al., 2018; Gagliano et al., 2018). From our previous work (Gagliano et al., 2018), we know that the 16S rRNA of the subspecies *M. harundinacea* is not positive (does not hybridize) to the three MX825 (a,b and c) probes available from literature. However, MSMX860 probe (Methanosarcinales) works well. In the samples analysed in this study, all the *Methanosaeta* cells were negative to MX825, but positive to MSMX860. Given this, and the results of our previous works, we were safe in driving out the discussion about *M. harundinacea*. Thus, the dominance of *Methanosaeta* cells in the total archaeal population was confirmed via FISH with ARC915, MSMX860 and MX825mix probes.

The hybridization carried out with ARC915 (total Archaea, red) and MSMX860 (*Methanosarcinales*, green) (stringency 45% of Formamide) gave a yellow signal (see image below), indicating that most of the archaeal population probably belongs to this *Methanosaeta* sp.

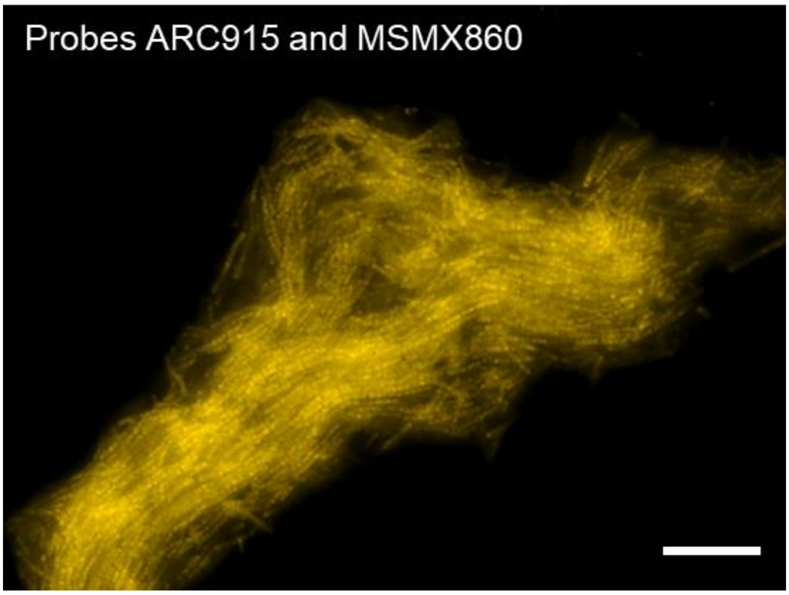

The peculiar *Methanosaeta* morphology was widespread, the only one detected when applying the two probes ARC915 and probe MSMX860. Moreover, a lot of microscopic characterization and work has been done on this sludge, and even the application of probes MG1200b (*Methanomicrobiales*) and MB311 (*Methanobacteriales*) did not show any significant result.

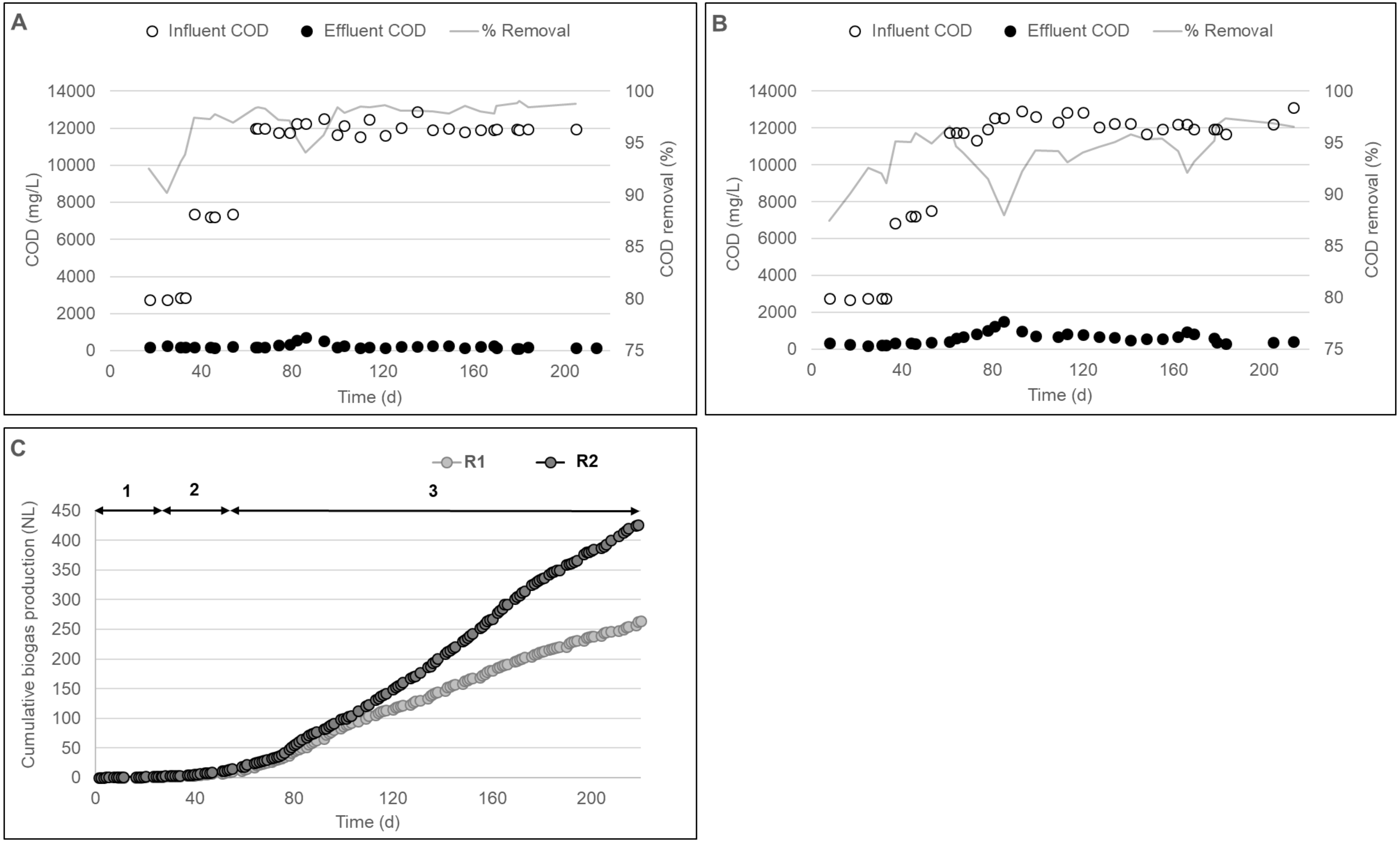

Fig. S2 - In A and B: Influent and effluent COD and the relative percentage of removal during the digestion time in reactor R1 and R2, respectively. In C: Cumulative biogas production of reactor R1 (grey) and reactor R2 (black), thoughout the digestion phases with increasing OLR (See Table 1).

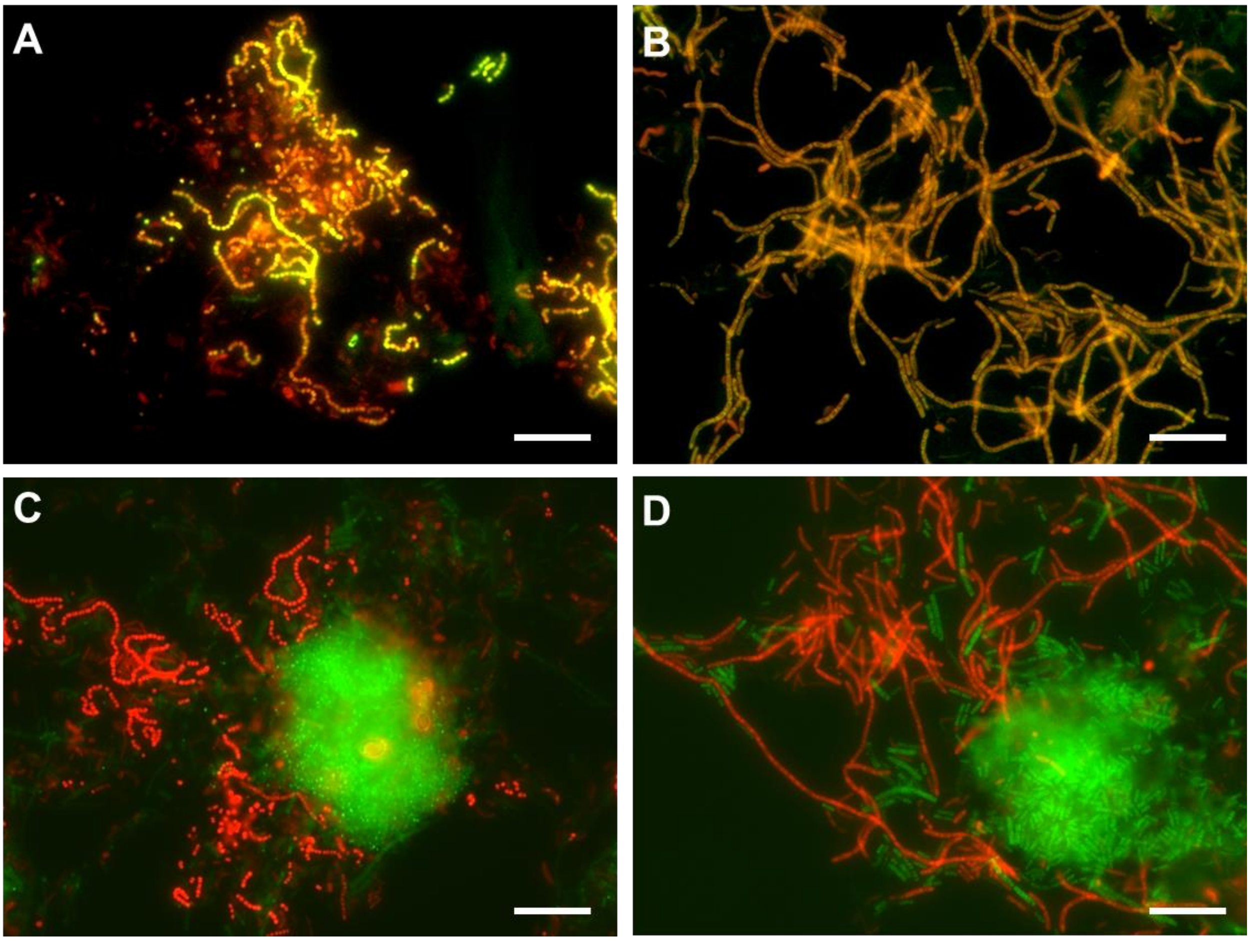

Fig.S3 – Epifluorescence images after FISH analysis of anaerobic granules. In A, *Streptococcus sp.* was detected in reactor R1 samples by using Strept probe. In B, filaments *Defluvitalea sp.* were further identified in reactor R2 with the probe LAC435 (green). In A and B, the EUB338mix probe (red) was applied together with specific probes (in green), resulting in a yellow signal.

In C and D, the EUB338mix probe (red) was applied together with ARC915 probe (in green) to show the association between the dominant filamentous bacteria and *Methanosaeta* clusters in reactor R1 (C) and reactor R2 (D). Scale bar is 10 µm.

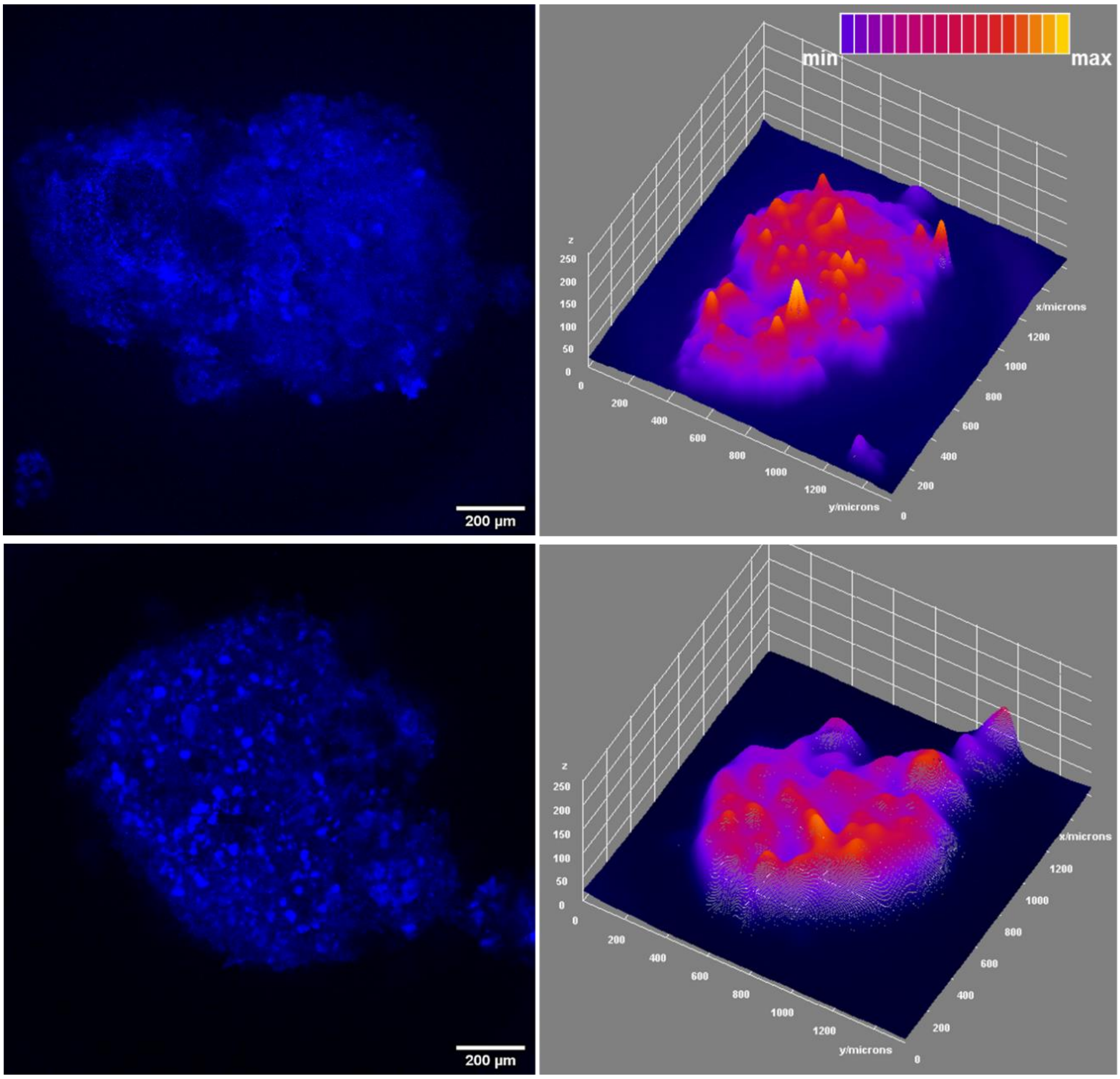

Fig.S4 – CLSM images (left) and the relative signal distribution (right) of F_{420} autofluorescence emission in high salinity granules, highlighting the presence of active *Methanosaeta* clusters throughout their structure.

CLSM analysis was carried out using a TCS SP5 (Leica, Wetzlar, Germany). CLSM datasets were recorded from granules in the multichannel mode taking advantage of F_{420} autofluorescence (Doddema and Vogels, 1978)

The FIJI software package (version1.51g, Wayne Rasband, NIH, Bethesda, MD, United States) was used to analyze/modify the CLSM stacks and to construct the signal distribution maps.

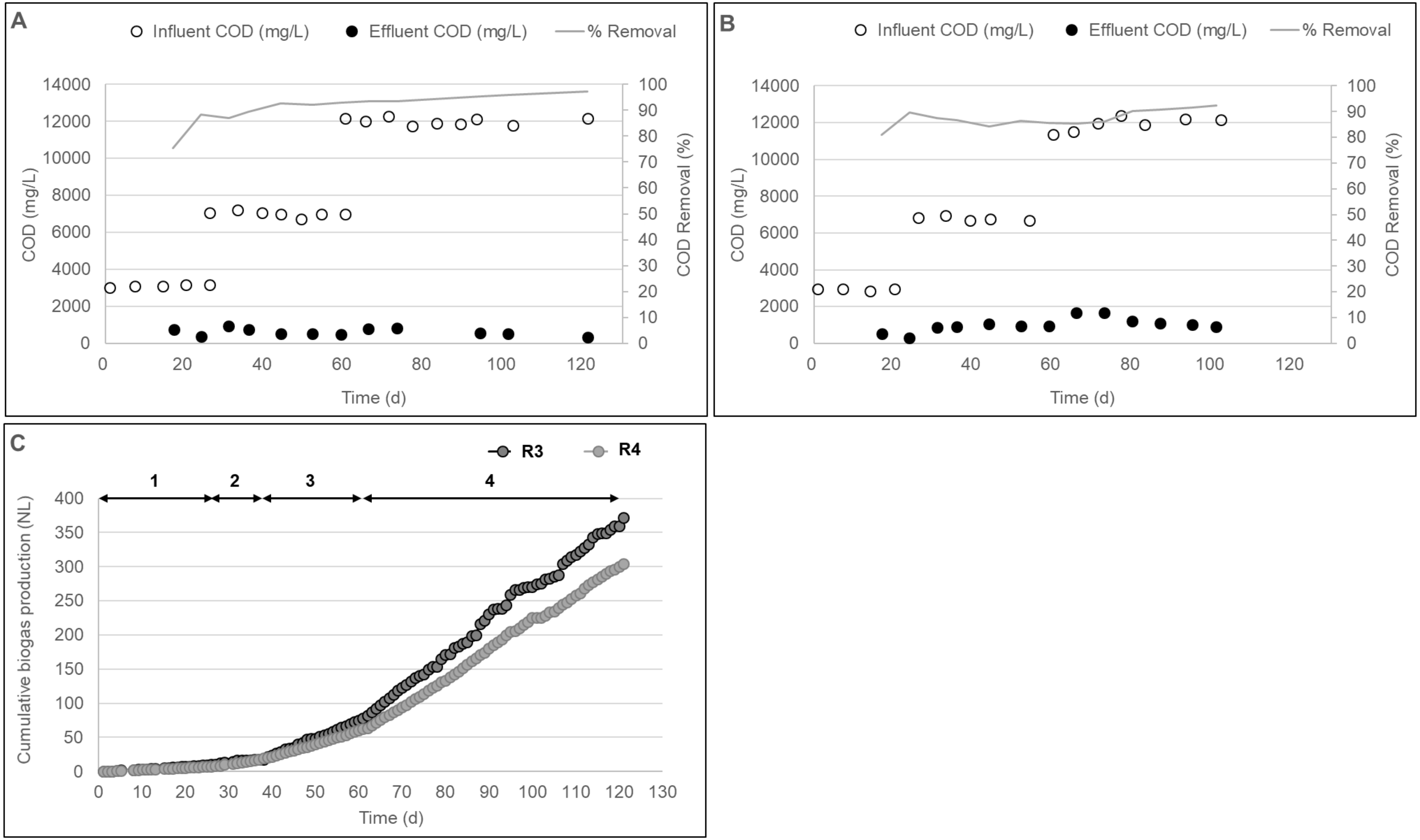

Fig. S5 - In A and B: Influent and effluent COD and the relative percentage of removal during the digestion time in reactor R3 and R4, respectively. In C: Cumulative biogas production of reactor R3 (black) and reactor R4 (grey), thoughout the digestion phases with increasing OLR (see Table 1).

Table S6 – Volatile fatty acids levels in reactors R3 and R4 along the digestion time. After day 75, in R4 the substrate leucine was substituted with glutamic acid.

		VFA concentration (mg/L)						
	**day**	*Acetate*	*Propionate*	*Isobutyrate*	*Butyrate*	*Isovalerate*	*Valerate*	*Isocaproate*
**R3**	*18*	224.1	116.3	14.8	0	24.1	0	16.4
	33	44.1	223.5	27.9	11.6	48.4	11.0	5.1
	46	21.5	137.5	23.9	0.0	28.6	0	1.1
	61	14.2	142.1	11.2	0.8	31.5	0	2.9
	75	139.3	287.2	5.5	1.1	51.4	0	3.7
	89	17.1	182.4	3.8	1.0	4.7	0	3.1
	106	78.2	234.8	4.6	0	28.1	0	3.4
**R4**	*18*	46.5	63.0	0	0	33.3	11.0	10.7
	33	231.0	170.4	7.0	1.2	90.8	26.0	2.8
	46	191.7	420.4	14.5	6.4	182.0	49.6	1.2
	61	24.2	314.2	12.9	0.8	142.4	5.2	3.1
	75	82.9	910.9	23.7	1.7	98.4	13.1	3.4
	89	49.2	664.2	15.0	0	6.4	7.4	1.3
	106	43.7	561.6	13.8	0	5.7	5.2	0.8

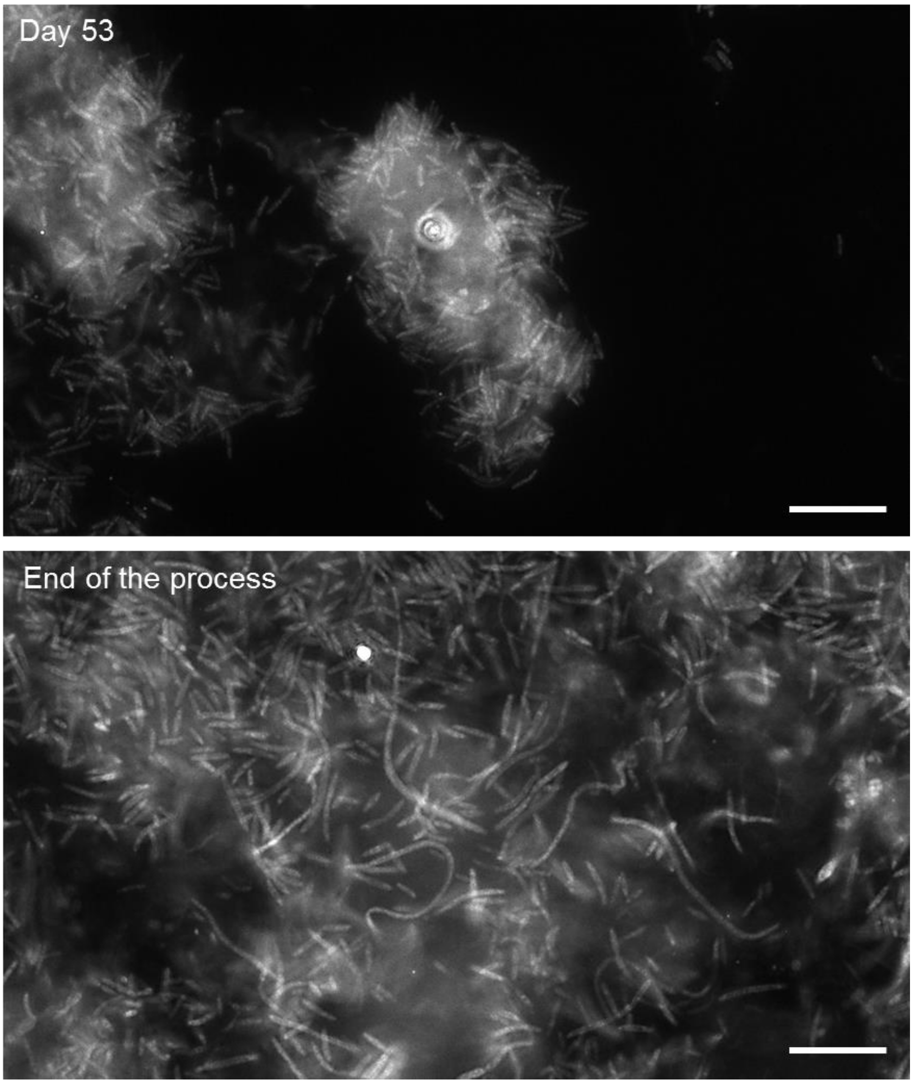

Fig. S6 – Epifluorescence images after FISH analysis of R3 reactor anaerobic granules at two different stages of the process, showing members of the family *Defluviitaleaceae* identified with the probe LAC435. Scale bar is 10 µm.

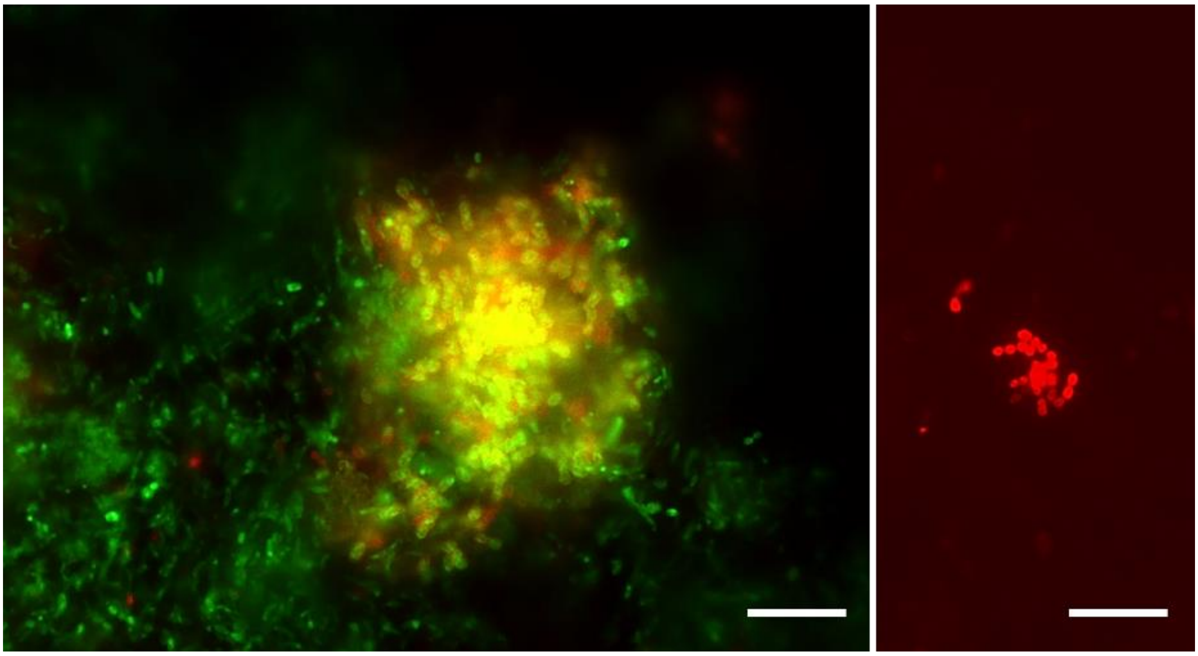

Fig.S7 – Epifluorescence images after FISH analysis of R4 reactor anaerobic biomass at the end of the process, showing *Enterococcus* ssp. identified with the probe LAB158 (in red). In green, total bacteria detected by EUB338 probe. Yellow signal corresponds to the double positivity to both probes. Scale bar is 10 µm.

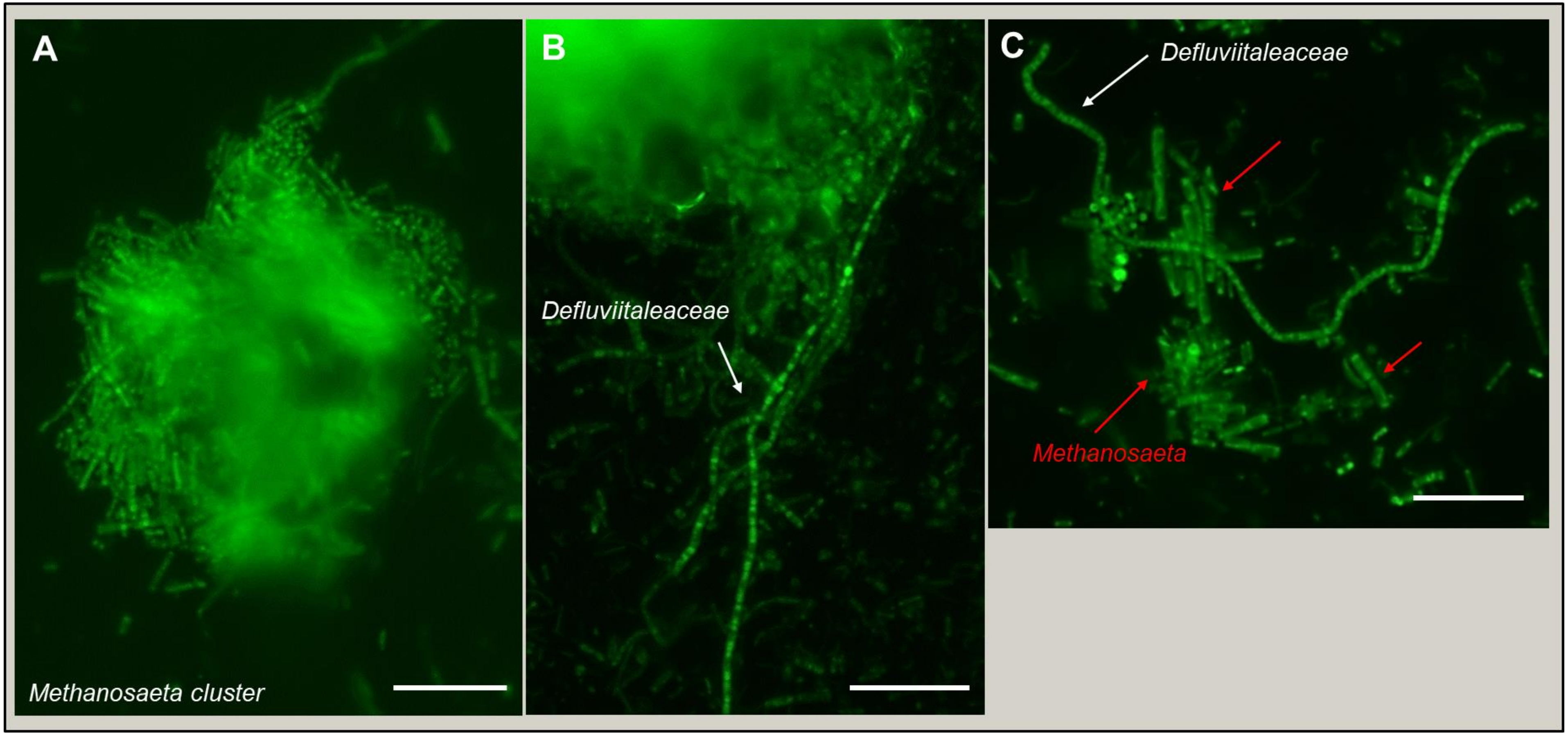

Fig. S8 – FITC protein staining of R2 granules, showing the positivity of *Methanosaeta* (A) and *Defluviitaleaceae* (B) outer layer. In C, the typical morphology of *Methanosaeta* (red arrow) and *Defluviitaleaceae* (white arrow) cells, previously highlighted by FISH (Fig. S2), are shown together. Size bar is 10 µm.

References

Amann, R., Binder, B., 1990. Combination of 16S rRNA-targeted oligonucleotide probes with flow cytometry for analyzing mixed microbial populations. Appl. enviromental Microbiol. 56, 1919–25.

Björnsson, L., Hugenholtz, P., Tyson, G.W., Blackall, L.L., 2002. Filamentous Chloroflexi (green non-sulfur bacteria) are abundant in wastewater treatment processes with biological nutrient removal. Microbiology 148, 2309–2318. https://doi.org/10.1099/00221287-148-8-2309

Caporaso, J.G., Kuczynski, J., et al, 2010. QIIME allows high throughput community sequencing data. Nat. Methods 7, 335–336. https://doi.org/10.1038/nmeth0510-335

Crocetti, G., Murto, M., Björnsson, L., 2006. An update and optimisation of oligonucleotide probes targeting methanogenic Archaea for use in fluorescence in situ hybridisation (FISH). J. Microbiol. Methods 65, 194–201.

Doddema, H.J., Vogels, G.D., 1978. Improved identification of methanogenic bacteria by fluorescence microscopy. Appl. Environ. Microbiol. 36, 752–754.

Edgar, R.C., 2010. Search and clustering orders of magnitude faster than BLAST. Bioinformatics 26, 2460–2461. https://doi.org/10.1093/bioinformatics/btq461

Gagliano, M.C., Braguglia, C.M., Gianico, a., Mininni, G., Nakamura, K., Rossetti, S., 2015. Thermophilic anaerobic digestion of thermal pretreated sludge: Role of microbial community structure and correlation with process performances. Water Res. 68, 498–509. https://doi.org/10.1016/j.watres.2014.10.031

Gagliano, M.C., Ismail, S.B., Stams, A.J.M., Plugge, C.M., Temmink, H., Van Lier, J.B., 2017. Biofilm formation and granule properties in anaerobic digestion at high salinity. Water Res. 121, 61–71. https://doi.org/10.1016/j.watres.2017.05.016

Gagliano, M.C., Neu, T.R., Kuhlicke, U., Sudmalis, D., Temmink, H., Plugge, C.M., 2018. EPS glycoconjugate profiles shift as adaptive response in anaerobic microbial granulation at high salinity. Front. Microbiol. 9, doi: 10.3389/fmicb.2018.01423.

Harmsen, H.J.M., Elfferich, P., Schut, F., Welling, G.W., 1999. A 16S rRNA-targeted probe for detection of lactobacilli and enterococci in faecal samples by fluorescent in situ hybridization. Microb. Ecol. Health Dis. 11, 3–12. https://doi.org/10.1080/089106099435862

Kong, Y., He, M., McAlister, T., Seviour, R., Forster, R., 2010. Quantitative fluorescence in situ hybridization of microbial communities in the rumens of cattle fed different diets. Appl. Environ. Microbiol. 76, 6933–6938. https://doi.org/10.1128/AEM.00217-10

Lücker, S., Steger, D., Kjeldsen, K.U., MacGregor, B.J., Wagner, M., Loy, A., 2007. Improved 16S rRNA-targeted probe set for analysis of sulfate-reducing bacteria by fluorescence in situ

hybridization. J. Microbiol. Methods 69, 523–528. https://doi.org/10.1016/j.mimet.2007.02.009

Manz, W., Amann, R., Ludwig, W., Wagner, M., Schleifer, K.H., 1992. Phylogenetic Oligodeoxynucleotide Probes for the Major Subclasses of Proteobacteria: Problems and Solutions. Syst. Appl. Microbiol. 15, 593–600. https://doi.org/10.1016/S0723-2020(11)80121-9

Meier, H., Amann, R., Ludwig, W., Schleifer, K.H., 1999. Specific oligonucleotide probes for in situ detection of a major group of gram-positive bacteria with low DNA G + C content. Syst. Appl. Microbiol. 22, 186–96. https://doi.org/10.1016/S0723-2020(99)80065-4

Quast, C., Pruesse, E., Yilmaz, P., Gerken, J., Schweer, T., Yarza, P., Peplies, J., Glöckner, F.O., 2013. The SILVA ribosomal RNA gene database project: Improved data processing and web-based tools. Nucleic Acids Res. 41. https://doi.org/10.1093/nar/gks1219

Raskin, L., Stromley, J.M., Rittmann, B.E., Stahl, D. a, 1994. Group-specific 16S rRNA hybridization probes to describe natural communities of methanogens. Appl. Environ. Microbiol. 60, 1232–40.

Stahl, D.A., Amann, R. 1991 Development and application of nucleic acid probes. In: Stackebrandt, E., Goodfellow, M. (Eds.), Nucleic acid techniques in bacterial systematics, John Wiley & Sons, Chichester, pp. 205–248.

Sudmalis, D., Gagliano, M.C., Pei, R., Grolle, K., Plugge, C.M., Rijnaarts, H.H.M., Zeeman, G., Temmink, H., 2018. Fast anaerobic sludge granulation at elevated salinity. Water Res. 128, 293–303. https://doi.org/https://doi.org/10.1016/j.watres.2017.10.038

Trebesius, K., Leitritz, L., Adler, K., Schubert, S., Autenrieth, I.B., Heesemann, J., 2000. Culture independent and rapid identification of bacterial pathogens in necrotising fasciitis and streptococcal toxic shock syndrome by fluorescence in situ hybridisation. Med. Microbiol. Immunol. 188, 169–175. https://doi.org/10.1007/s004300000035

Wang, Q., Garrity, G.M., Tiedje, J.M., Cole, J.R., 2007. Naive Bayesian classifier for rapid assignment of rRNA\nsequences into the new bacterial taxonomy. Appl. Environ. Microbiol. 73, 5261–5267. https://doi.org/10.1128/AEM.00062-07
